# Quantitative Analysis of Adventitious Root Growth Phenotypes in Carnation Stem Cuttings

**DOI:** 10.1371/journal.pone.0133123

**Published:** 2015-07-31

**Authors:** Virginia Birlanga, Joan Villanova, Antonio Cano, Emilio A. Cano, Manuel Acosta, José Manuel Pérez-Pérez

**Affiliations:** 1 Instituto de Bioingeniería, Universidad Miguel Hernández, Elche, Spain; 2 Departamento de Biología Vegetal (Fisiología Vegetal), Universidad de Murcia, Murcia, Spain; 3 Research and Development Department, Barberet & Blanc S.A., Puerto Lumbreras, Spain; Instituto de Biología Molecular y Celular de Plantas, SPAIN

## Abstract

Carnation is one of the most important species on the worldwide market of cut flowers. Commercial carnation cultivars are vegetatively propagated from terminal stem cuttings that undergo a rooting and acclimation process. For some of the new cultivars that are being developed by ornamental breeders, poor adventitious root (AR) formation limits its commercial scaling-up, due to a significant increase in the production costs. We have initiated a genetical-genomics approach to determine the molecular basis of the differences found between carnation cultivars during adventitious rooting. The detailed characterization of AR formation in several carnation cultivars differing in their rooting losses has been performed (i) during commercial production at a breeders’ rooting station and (ii) on a defined media in a controlled environment. Our study reveals the phenotypic signatures that distinguishes the bad-rooting cultivars and provides the appropriate set-up for the molecular identification of the genes involved in AR development in this species.

## Introduction

Carnation (*Dianthus caryophyllus* L.) is, after rose, the most important species on the worldwide market of cut flowers, with a yearly sales volume of almost 200 million plants [1]. Current ornamental breeding and production depends largely on rapid multiplication of elite clones, production of healthy and disease-free plants and faster introduction of novel cultivars.

Commercial carnation cultivars are propagated from terminal stem cuttings with 4–6 pairs of leaves [2]. Once separated from the mother plant, the cuttings must remain deprived of the root during cold storage [3, 4]. Rooting is a very sensitive and highly energy-demanding process affected by complex interactions between sucrose and hormone levels [5, 6]. Rooted cuttings are then transferred to hardening chambers before transplanting them to production fields. The production of young plantlets is frequently hampered by minimal adventitious root (AR) formation from stem cuttings, which has a strong genetic dependency and which leads to production losses in certain carnation cultivars [7, 8]. The problem has been partly overcome by optimizing the storage of fresh cuttings at low temperature [4, 9]. Insufficient rooting extends the production time, causes waste of resources and leads to production losses in certain cultivars. For some of the new cultivars that are being developed by ornamental breeders, poor AR formation limits its commercial scaling-up, due to a significant increase in the production costs. Hence production economics and ecology would tremendously benefit from improved growth characteristics of stem cuttings as well as from reduced high-cost and energy-demanding hardening treatments.

In our attempt to determine the genetic basis of AR development in carnation, we selected several good-rooting and bad-rooting cultivars for a detailed characterization of their root system over time. To maximize the contribution of endogenous (genetic) factors responsible for the differences in AR formation between cultivars, phenotyping was performed on fresh stem cuttings without the aid of exogenous auxin. The entire root system developed at the base of the stem cutting was studied at regular time-intervals using a non-invasive imaging method on a controlled environment. In addition, phenotyping of AR formation was also performed on a breeders’ rooting station which allowed us to define a qualitative scale of non-overlapping stages for visual assignment of rooting performance. Morphological and ecophysiological data from the shoot region of the stem cutting was also scored. Our results indicate that most of the differences observed between bad-rooting and good-rooting cultivars are caused by a delay in AR initiation from the base of the stem cutting, a reduced number of root primordia, and/or a slow growth rate of primary and secondary ARs in the bad-rooting cultivars. This study will set the bases for the molecular identification of the genes involved in AR formation in this species that will help to establish a marker-assisted selection approach to select for improved AR performance in current carnation breeding programs.

## Materials and Methods

### Plant material

From stored data about rooting losses scored in 132 commercial carnation cultivars that were vegetatively propagated at Barberet & Blanc (http://www.barberet.es) between 2011 and 2013, we selected 10 cultivars for further studies (S1 Table). Stem cuttings from the cultivars used in this work are available upon request. Due to organizational issues, about 110 stem cuttings were pinched from several mother plants of each cultivar by skilled operators at noon on 29^th^ April 2013 (batch 1) and 20^th^ May 2013 (batch 2). All the mother plants had been grown in the same glasshouse under environmental conditions at 37°34´50´´ N, 1°46´35´´ W and 395 altitude (Puerto Lumbreras, Murcia, Spain). Stem cutting lengths and fresh weights were measured to discard the outliers in each cultivar (Table 1). Ninety of the most representative stem cuttings per cultivar were kept for further analyses: (i) stem cutting ecophysiology and morphometric analysis, (ii) adventitious rooting in soil plugs and (iii) *in vitro* adventitious rooting.

**Table 1:** Gross morphology and some ecophysiological traits of unrooted carnation stem cuttings.

Cultivar code	Cutting length (cm)^a^	Cutting fresh weight (g)^a^	Cutting water content (g)^b^	Cuticular evaporation (g/day) ^b^	Leaf area (cm^2^)	Grade of succulence (g/cm^2^)	Specific leaf area (cm^2^/g)	Leaf dry matter content (g/g)
13-78-1 MFC	12.70 ± 1.06	1.53 ± 0.28	1.55 ± 0.22 D	0.20 ± 0.03 E	4.60 ± 0.50 C	0.024 ± 0.002 BCD	147.97 ± 8.30 ABC	0.126 ± 0.008 AB
189 R	15.81 ± 1.25	2.32 ± 0.42	2.30 ± 0.27 BC	0.38 ± 0.09 AB	6.21 ± 1.91 BC	0.024 ± 0.003 BCD	172.33 ± 16.26 A	0.110 ± 0.010 CD
2000 MFJ 7	14.19 ± 0.90	2.06 ± 0.33	2.03 ± 0.23 CD	0.50 ± 0.09 A	5.05 ± 0.94 C	0.022 ± 0.001 D	167.68 ± 12.03 AB	0.120 ± 0.009 ABC
2003 R 8	16.12 ± 1.26	2.93 ± 0.59	2.74 ± 0.31 B	0.36 ± 0.10 BC	5.00 ± 1.20 C	0.026 ± 0.001 ABC	145.57 ± 13.44 ABC	0.117 ± 0.007 BCD
2007 R 32	17.92 ± 1.76	2.50 ± 0.61	2.42 ± 0.34 BC	0.47 ± 0.11 AB	6.40 ± 0.62 BC	0.023 ± 0.002 CD	163.84 ± 15.46 AB	0.119 ± 0.007 BCD
2101–02 MFR	19.29 ± 1.32	2.78 ± 0.47	2.74 ± 0.34 B	0.23 ± 0.11 CDE	7.91 ± 0.86 AB	0.026 ± 0.001 ABC	121.95 ± 3.95 C	0.136 ± 0.006 A
2441–7 R	15.96 ± 1.24	3.83 ± 0.84	4.47 ± 0.59 A	0.36 ± 0.12 BCD	5.75 ± 0.83 BC	0.029 ± 0.003 A	144.10 ± 10.82 BC	0.107 ± 0.007 CD
3002 P	16.35 ± 1.64	2.37 ± 0.39	2.45 ± 0.30 BC	0.38 ± 0.10 AB	5.40 ± 0.67 BC	0.027 ± 0.001 AB	163.73 ± 15.08 AB	0.103 ± 0.007 C
N 576 B	20.70 ± 1.40	4.17 ± 0.85	3.91 ± 0.84 A	0.23 ± 0.06 DE	10.11 ± 2.34 A	0.030 ± 0.003 A	126.30 ± 14.15 C	0.118 ± 0.010 CD
R 667 FJ FOR	15.86 ± 1.15	2.58 ± 0.42	2.65 ± 0.21 B	0.44 ± 0.07 AB	4.83 ± 0.95 C	0.024 ± 0.002 BCD	160.91 ± 12.26 AB	0.115 ± 0.005 BCD

A minimum of ^a^100 or ^b^10 stem cutting samples were analyzed. Different letters indicate significant differences (*P* < 0.01) between the cultivars.

### Stem cutting ecophysiology

Some ecophysiological stem cutting and leaf traits were measured as described in [10]. Ten cuttings and five full-developed leaves per cultivar were used for the measurements. Cuttings and leaves were rehydrated by full immersion in tap water for 24h and later they were gently dried with soft-paper before being used for the different determinations. Cutting water saturated weight (CWSW) was estimated as the cutting weight after full rehydration. Then cuttings were kept in a chamber at 4°C and 75% relative humidity (RH) with no further rehydration, and their weight loses were daily monitored. Cuticular evaporation (Ecut) was estimated by measuring the water loses of rehydrated cuttings after one day of being transferred to the chamber, and considered as a linear function. Finally, for cutting dry weight (CDW) determination, the cuttings were kept in an oven at 60°C for 24 hours. To estimate leaf area (LA), images of individual leaves were obtained using a flat-bed scanner (300 dpi). Scanned images were then batch-processed with ImageJ software with available plug-in macros [11]. Leaf water saturated weight (LWSW), leaf dry weight (LDW) and leaf water content (LWC) were obtained following the same procedure described above for the cuttings. Grade of succulence (GS) is the ratio between leaf water content and two-sided leaf area, *GS* = *LWC*/2 *xLA* expressed as g/cm^2^. Specific leaf area (SLA) is the one-sided leaf area divided by the leaf dry weight, *SLA* = *LA/LDW* expressed as cm^2^/g. Leaf dry matter content (LDMC) is the ratio between leaf dry weight and leaf water saturated weight, *LDMC* = *LDW/LWSW*.

### Adventitious rooting in soil plugs

For each cultivar, 30 freshly-harvested stem cuttings were immediately submerged for 15 h in an aqueous fungicide solution (1 g l^-1^ benomyl) and without exogenous auxin treatment, at 15 ± 2°C and 80% RH in dim light (4 μmol m^-2^ s^-1^ of average photosynthetic photon flux density [PPFD]). Afterwards, stem cuttings were individually planted in peat-perlite (90−10 v/v) substrate trays of 294 truncated pyramid plugs (2.5 × 2.5 × 4.0 cm; 16 cm^3^) under glasshouse conditions at Barberet & Blanc’s rooting station, as described previously [3]. Water, fertilizers and adequate phytosanitary treatments were periodically applied by skilled operators according to standard procedures for homogeneous production of commercial rooted cuttings.

### In vitro adventitious rooting

**Fig 1:**
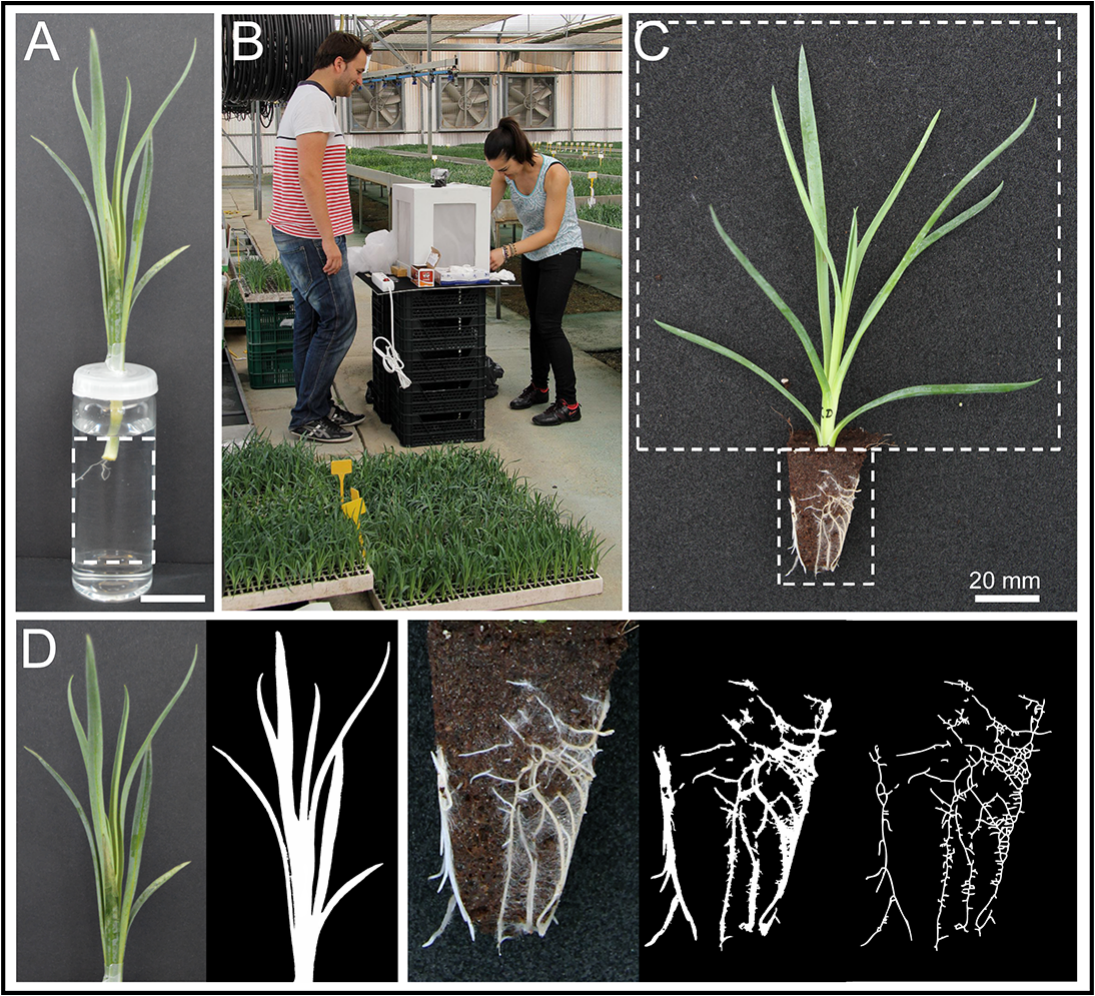
Morphological characterization of carnation stem cutting phenotypes. (A) A representative image of a stem cutting grown *in vitro* for 17 days. (B) Portable photographic bench used for image acquisition of stem cuttings grown in soil plugs at the rooting station. (C) A representative image of a stem cutting grown in soil plugs for 27 days. Dotted lines indicate the area of the image used for the morphometric analysis. (D) Image segmentation files obtained with the Gia Roots software, as described in Materials and Methods.

Thirty stem cuttings of each cultivar were immediately wrapped in plastic bags after pinching and were stored in a cold chamber at 5 ± 2°C, 60% RH and complete darkness until they were planted (1 month). Prior to planting, the base of the stem cuttings was washed in 70% (v/v) ethanol for 30 s, surface-sterilized in 3% (w/v) sodium hypochlorite for 10 min, and rinsed thoroughly with sterile distilled water (5 times). Each stem cutting was planted on a 40 ml glass scintillation vial (Fig 1A) containing 35 ml of half-strength Murashige and Skoog (MS) basal salt medium (Duchefa, The Netherlands), 50 mg l^-1^ ampicillin (Duchefa, The Netherlands), and 0.5 g l^-1^ 2-(*N*-morpholino)ethanesulfonic acid (MES; Duchefa, The Netherlands), pH 5.8. Plant cultures were grown on a Panasonic MLR-352 growth chamber set at 22 ± 2°C, 70% RH and a 16:8 (light:dark) photoperiod with average PPFD of 40 μmol m^-2^ s^-1^.

### Sample collection and image analysis

For scoring adventitious rooting at the rooting station, a minimum of three cuttings per cultivar were imaged 13 days after planting (DAP). All the remaining stem cuttings (up to 30) were also imaged 20 and 27 DAP. Stem cutting pictures were taken from the four sides of the soil plug using a Canon 60D camera with a Canon EF-S 17-85mm f/4-5.6 IS USM lens at a resolution of 5184 × 3466 pixels, and saved as an RGB color image in jpeg format. To minimize variations due to sunlight quality during the day, a portable photographic bench was used and the images were taken at the glasshouse between 11 am—1 pm (Fig 1B). For the morphometric analysis of stem cutting images (Fig 1C), individualized vegetative (2800 × 2800 pixels) or soil plug (550 × 1100 pixels) regions were batch-imported into the Gia Roots software [12]. After scale calibration and grayscale conversion, stem cutting or soil plug images were segmented using the global thresholding method (Fig 1D). Eventually, threshold was manually adjusted at each image batch (cultivar and time) to maximize object identification (leaves or roots). *In vitro* adventitious rooting was imaged periodically at 13, 15, 17, 20, 22, 24, 27 and 29 DAP. Pictures were taken using a Sony Cyber-shot DSC-H3 camera (Sony Corporation, Tokyo, Japan) at a resolution of 3264 × 2448 pixels, and saved as an RGB color image in jpeg format. A defined region in each image (1000 × 1100 pixels) containing the rooting region was used for image segmentation with the Gia Roots software as described above. All 19 root system architectural traits established previously were initially selected and were computed directly from the image mask or from the skeleton of the image mask as described elsewhere [12]. Raw measurements were exported to Excel spreadsheets for data analysis. Raw data files are available upon request.

### Statistical analysis

Descriptive statistics (mean, standard deviation [SD], maximum and minimum, etc.) were calculated for samples taken at each stem cutting by using the StatGraphics Centurion XV software (StatPoint Technologies, Inc. Warrenton, VA USA) and SPSS 21.0.0 (SPSS Inc., Chicago, IL, USA) programs. Data outliers were identified based on aberrant SD values and excluded for posterior analyses. One-sample Kolmogorov-Smirnov tests [13] were performed to analyze the goodness-of-fit between the distribution of the data and a given theoretical distribution as previously described [14]. The differences between the data groups were analyzed by t test (*P* < 0.05) when only two groups were compared. To compare the data for a given variable, we performed multiple testing analyses with the ANOVA F-test or the Fisher’s LSD (Least Significant Differences) methods [15]. Non-parametric tests were used when necessary. In that case median was used instead of mean. The differences between the data groups were analyzed by Mann-Whitney U test (*P* < 0.05) when only two groups were compared. In the other cases, data were subjected to Kruskal-Wallis test (*P* < 0.05). Correlations were studied using Pearson product-moment correlation coefficient (Pearson’s r) [16]. Principal component analysis was used to reduce the dimensions of our sets of variables as previously described [14].

## Results

### Stem cutting losses during adventitious rooting differ between carnation cultivars

Rooted stem cuttings of commercial quality are characterized by the presence of 30 − 40 well developed roots of 1 − 9 cm length and by a healthy and homogeneous shoot apex at one month after planting (Fig 1C). Rooted cuttings of insufficient quality are manually removed before commercialization, which increases labor costs. Stem cutting losses on a collection of 132 commercial carnation cultivars grown at the Barberet & Blanc’s rooting station between 2011 and 2013 ranged between 0.83 ± 0.89% and 13.54 ± 8.59% (S1 Fig), with significant lower values in spray cultivars (3.93 ± 2.25%) than in standard cultivars (4.72 ± 1.97%).

To initiate a quantitative description of rooting performance in carnation stem cuttings, we chose eight cultivars displaying extreme and contrasting values of rooting losses and two cultivars representative of the mean behavior for this trait within the studied population (S1 Table). We found that lengths and fresh weights of the stem cuttings collected for the rooting experiment were positively correlated (r = 0.687; *P* < 0.005), and that both parameters differed considerably among the studied cultivars, ranging between those in the *13-78-1 MFC* and the *N 576 B* cultivars (Table 1). Also, we observed a strong and significant effect of batch and cultivar type (spray or standard) on the size of the stem cuttings collected. At this point, we could not rule out whether the stem cutting size differences observed between batches are due to differences between mother plant age or due to glasshouse conditions during their growth.

### Morphometric characterization of the stem cutting phenotype in representative carnation cultivars

**Fig 2:**
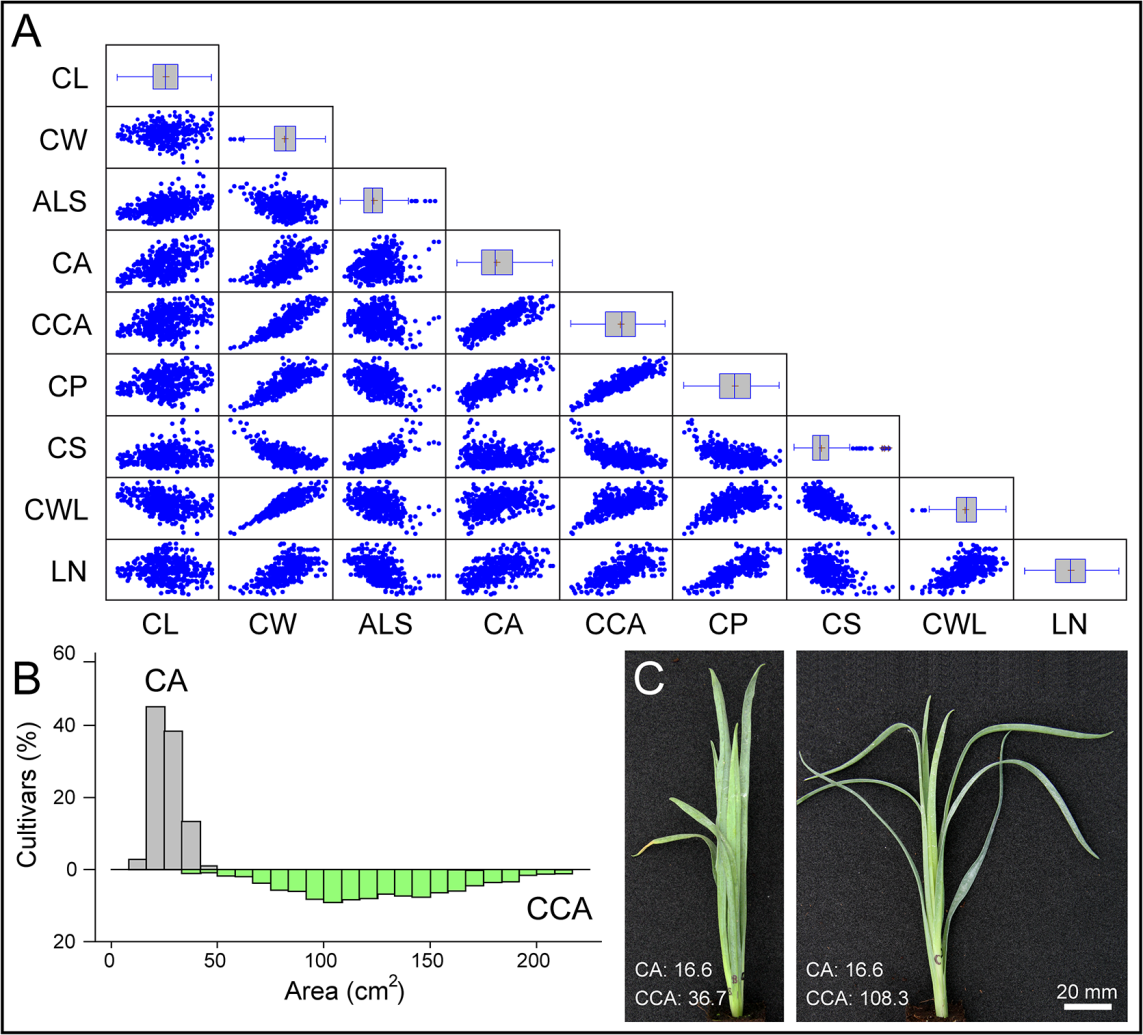
Stem cutting morphology. (A) Scatter plots of vegetative stem cutting parameters as defined in Table 2. Box-plots of each parameter are also represented. (B) Histograms for CA and CCA parameters. (C) Two stem cutting images displaying similar CA values but that differ in their CCA values (in cm^2^). Images were obtained as described in Materials and Methods at 20 DAP.

For the quantitative phenotyping of stem cutting morphology, we measured nine parameters in the four sides of each stem cutting at 20 and 27 DAP (Table 2). We found significant correlations for most of the parameters measured (Fig 2A) although *r* ^2^ values between any two variables rarely exceed 0.65 (S2 Table). For each parameter we performed multifactorial ANOVA considering cultivar type (spray or standard), rooting performance, batch experiment and DAP. CL was found significantly influenced by batch experiment and was hardly modified for a given cultivar (S2A Fig). ALS was mainly influenced by cultivar type (S2B Fig) thus spray cultivars displayed narrower leaves (2.74 ± 0.02 mm) than standard cultivars (3.26 ± 0.02 mm). In the studied population, CA variation range was narrow (38.81 cm^2^) compared to that of CCA (250.50 cm^2^) (Fig 2B). Hence, stem cuttings with similar CA values presented striking differences in their CCA values (Fig 2C), which could be explained by changes in the insertion angle of older leaves during the experiment (see below).

**Table 2:** Morphometric and ecophysiological parameters studied.

**Stem cutting morphometry**	**Symbol**	**Calculated in Gia Roots as**	**Unit**
Cutting length	CL	Network depth (Ndepth)	cm
Cutting width	CW	Network width (Nwidth)	cm
Average leaf section	ALS	Average root width (Width)	mm
Cutting area	CA	Network area (NwA)	cm^2^
Convex cutting area	CCA	Convex area (ConvA)	cm^2^
Cutting perimeter	CP	Perimeter (Perim)	cm
Cutting solidity	CS	Network solidity	cm^2^/cm^2^
Cutting width-to-length ratio	CWL	Network width to depth ratio	cm/cm
Leaf number	LN	nd	
**Stem cutting ecophysiology**		**Estimated as**	**Unit**
Cutting water content	CWC	CWSW-CDW	g
Cuticular evaporation	Ecut	This paper	g/day
Leaf area	LA	This paper	cm^2^
Grade of succulence	GS	LWC/2×LA	g/cm^2^
Specific leaf area	SLA	LA/LDW	cm^2^/g
Leaf dry matter content	LDMC	LDW/LWSW	g/g
**Root system parameters**	**Symbol**	**Calculated in Gia Roots as**	**Unit**
Rooting stage	RSG	nd	
Average root diameter	ARD	Average root width (Width)	mm
Root depth	RD	Network depth (Ndepth)	cm
Root width	RW	Network width (Nwidth)	cm
Root length	RL	Network length (Nlen)	cm
Root area	RA	Network area (NwA)	cm^2^
Convex root area	CRA	Convex area (ConvA)	cm^2^
Root perimeter	RP	Perimeter (Perim)	cm
Root width-to-depth ratio	RWD	Network width to depth ratio	cm/cm
Root length distribution	RLD	Network length distribution (Ldist)	
Root solidity	RS	Network solidity	cm^2^/cm^2^
Maximum number of roots	MXR	Maximum number of roots (MaxR)	

Nd: not determined.

**Fig 3:**
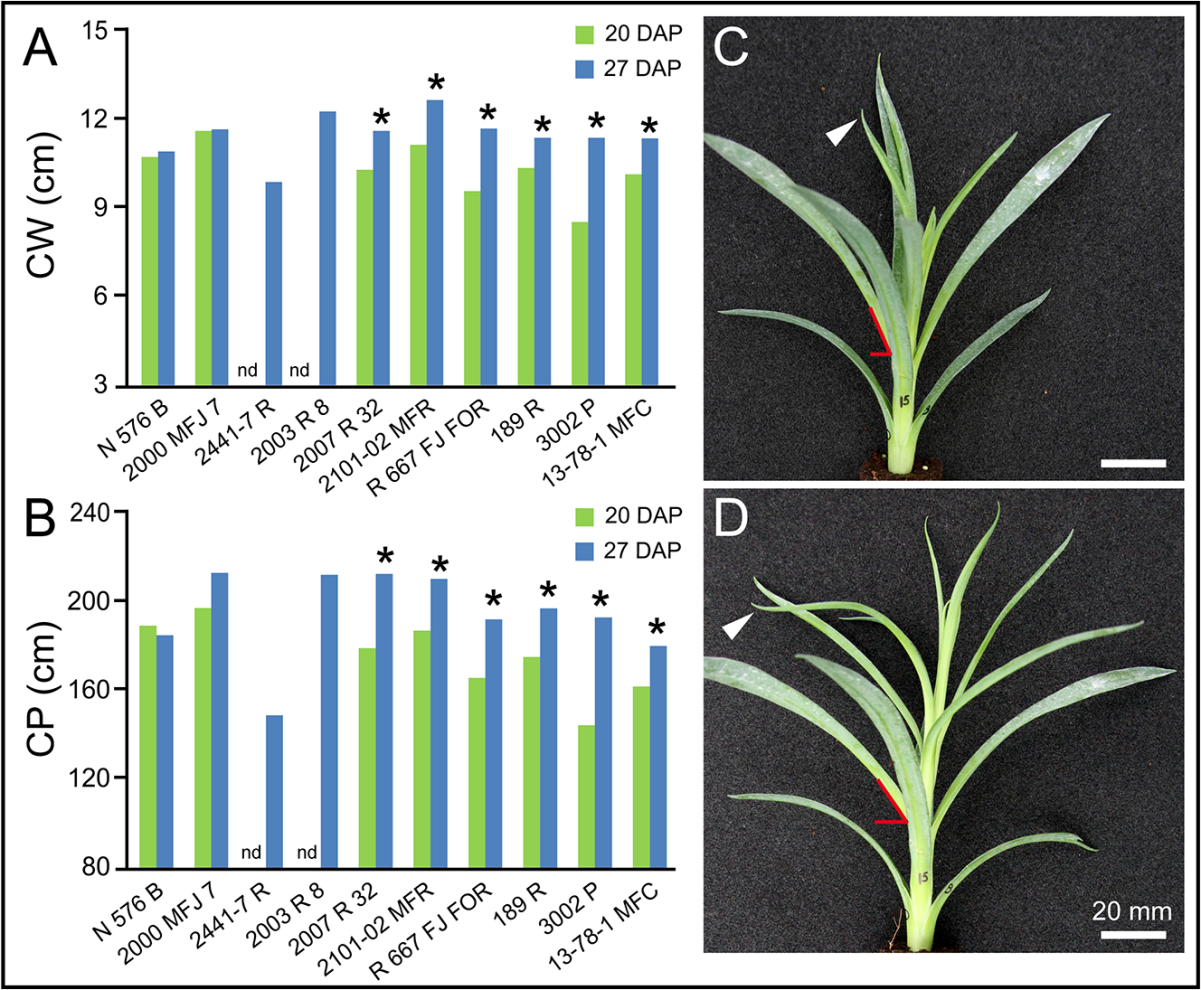
Stem cutting morphology of carnation cultivars. Average stem cutting (A) width and (B) perimeter values are shown for the studied cultivars. Asterisks indicate significant differences (*P* < 0.05) over time for a given cultivar. Nd: not determined. (C-D) A representative stem cutting imaged (C) 20 and (D) 27 DAP. White arrowhead points to the same leaf. Red lines are drawn to highlight leaf angles. Images were obtained as described in Materials and Methods.

Differences in CW, CP, CA, CCA and LN values differed significantly in most cultivars between 20 and 27 DAP, with the exception of cultivars *2441–7 R* and *2003 R 8*, which otherwise rooted poorly and no stem cutting data was collected for these two cultivars at 20 DAP (see next section). The overall differences in CW observed between 20 and 27 DAP (Fig 3A) are likely caused by the changes in the insertion angle of the outer (older) leaves of the stem cutting during the experiment (Fig 3C and 3D). On average, stem cuttings held 1.39 ± 0.40 more leaves at 27 DAP compared to those at 20 DAP. Taking CP as an unbiased descriptor of stem cutting size, differences in CP between 27 and 20 DAP for a given sample were considered an indirect estimate of stem cutting growth. Consequently, CP values increased between 13.1% in *2000 MFJ 7* to 31.3% in *3002 P* (Fig 3B). Stem cutting growth was mainly restricted to the younger leaves. Similar trends were also observed for CA and CCA (S2C and S2D Fig). We concluded that stem cutting morphology and stem cutting growth were heterogeneous among the selected carnation cultivars and significantly changed during rooting.

### Stem cutting ecophysiology in cultivated carnation

**Fig 4:**
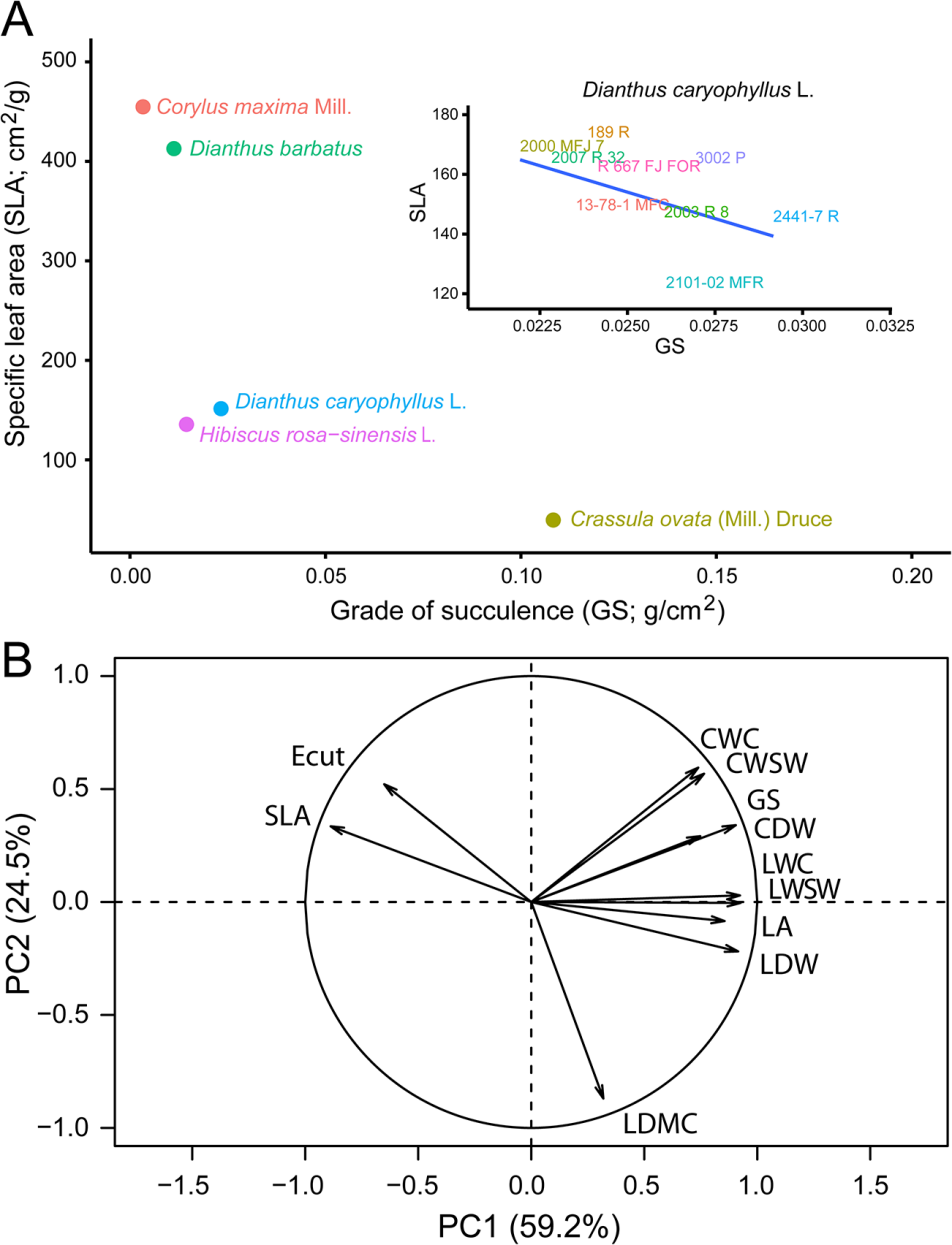
Stem cutting ecophysiology. (A) Representative distribution of species according to SLA and GS average values. Insert: Distribution of the *D*. *caryophyllus* cultivars studied in this work. (B) Principal component analysis of ecophysiological parameters. Graphical representation of PC1 and PC2 are shown.

Once detached from the mother plant, stem cuttings quickly start losing water. Hence, we studied some of their ecophysiological traits related to water economy. Differences in measured traits were observed between the studied cultivars (Table 1). Cutting water content (CWC) values ranged from 1.55 ± 0.22 g (*13-78-1 MFC*) to 4.47 ± 0.59 g (*2441–7 R*). Water-loss rate, represented by cuticular evaporation (E_cut_), was highest in the *2000 MFJ 7* cultivar (0.50 ± 0.09 g/day), while the lowest rate was observed in the *13-78-1 MFC* cultivar (0.20 ± 0.03 g/day). Graphical representation of specific leaf area (SLA) and grade of succulence (GS) for the studied carnation cultivars are shown in Fig 4A as compared with other ornamental species. While xerophytic species displayed low SLA and high GS values (*C*. *ovata*), all carnation cultivars studied so far clustered together with intermediate SLA values and low GS. A principal component (PC) analysis on 11 ecophysiological parameters was performed (Fig 4B). The first two components explained the 83.7% of the variance. PC1 seems to be related to water performance, showing a positive relationship with water retention (CWC, LWC, LWSW) and a negative relationship with water evaporation (SLA and Ecut), while PC2 is related to the water/dry weight ratio (LDMC).

### Adventitious rooting in cultivated carnation

Previous experiments showed that under the assay conditions at Barberet & Blanc’s rooting station almost no adventitious roots emerged in the base of the stem cuttings before 9 DAP, regardless of cultivar, auxin treatment or cold-storage period [3, 17]. Hence, sample images to characterize adventitious rooting in the selected cultivars were taken from freshly harvested stem cuttings grown for 13, 20 and 27 DAP in soil plugs without auxin treatment to maximize the differences between cultivars due to endogenous factors (see Materials and Methods).

**Fig 5:**
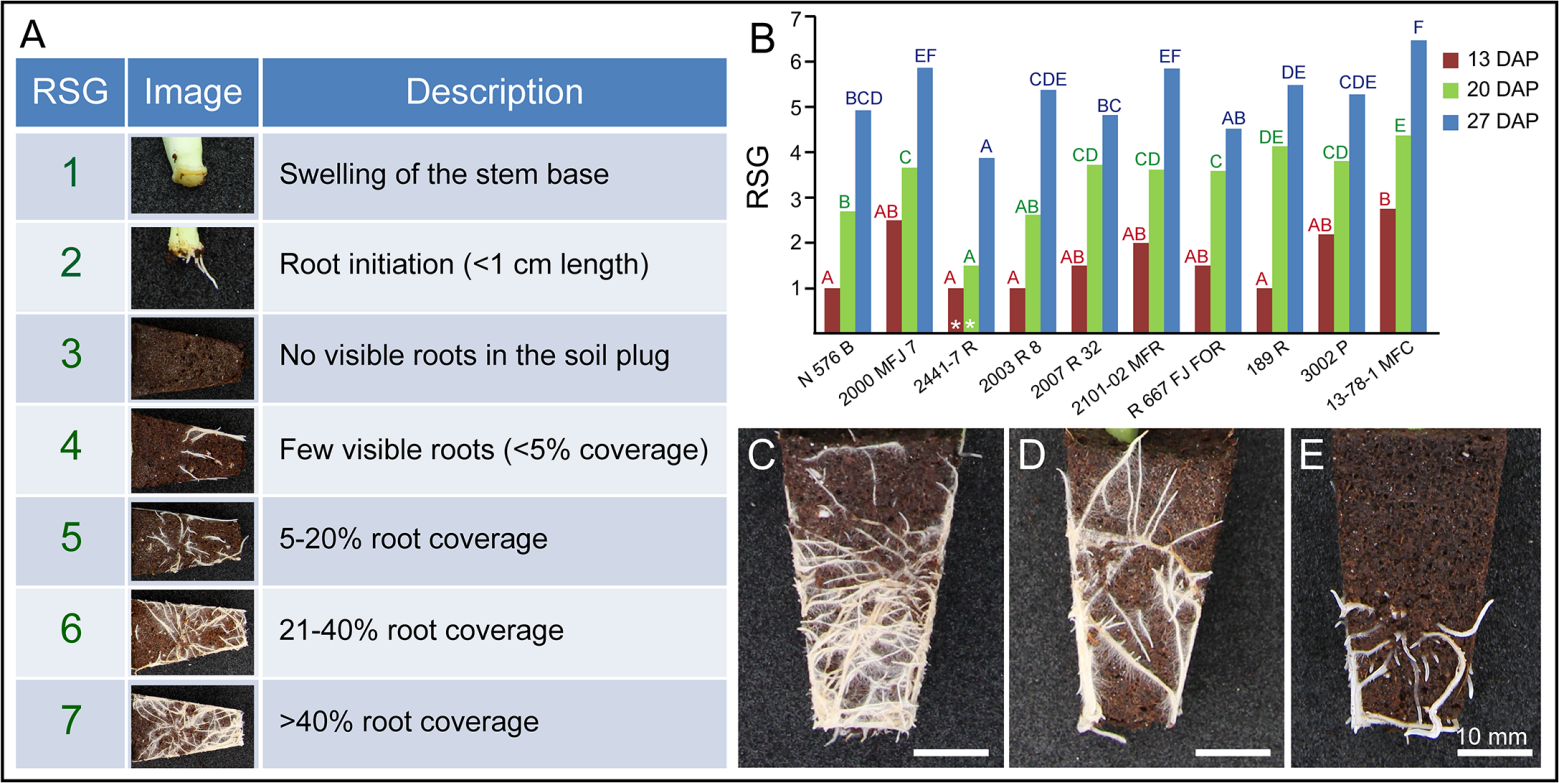
Qualitative description of adventitious rooting in carnation stem cuttings grown in soil plugs. (A) Rooting stages (RSG) based on visual assignment. Only samples from stage 3 onwards were used for later image analysis. (B) Graphic representation of average RSG values in different carnation cultivars over time. White asterisks highlight non-significant differences between the indicated time points for a given cultivar. Different letters indicate significant differences (*P* < 0.01) over cultivars for a given time. Representative soil plug images of adventitious rooting in (C) a good-rooting cultivar (*13-78-1 MFC*), (D) an intermediate-rooting cultivar (*2007 R 32*) and (E) a bad-rooting cultivar (*2441–7 R*). Images were obtained as described in Materials and Methods at 27 DAP.

Based on previous knowledge of skilled personnel at the Barberet & Blanc’s rooting station, we visually defined seven rooting stages representing the different adventitious root phenotypes observed (Fig 5A). In stages 1 and 2, the stem cuttings were easily isolated from the soil plug and no further morphometric analysis was conducted on these cuttings. Images for the morphometric analysis of the root system were obtained from stage 3 onwards. Stage 7 was assigned to stem cutting images where more than 40% of the soil plug was covered by roots and that represented stem cuttings of commercial quality. The cultivars studied here showed remarkable differences in rooting performance at different DAP as estimated by the RSG parameter (Fig 5B). At 13 DAP, only the *13-78-1 MFC* cultivar displayed differentially enhanced rooting performance. At 20 DAP, the cultivars clustered in five groups with *2441–7 R* and *13-78-1 MFC* as the ones displaying the most contrasting rooting performance. At 27 DAP, the differences in rooting performance between most of the studied cultivars diminished. Interestingly, some cultivars that initially showed poor rooting performance (such as *N 576 B* and *2003 R 8*) behaved as good-rooting cultivars at the end of the experiment (Fig 5B–5E).

**Fig 6:**
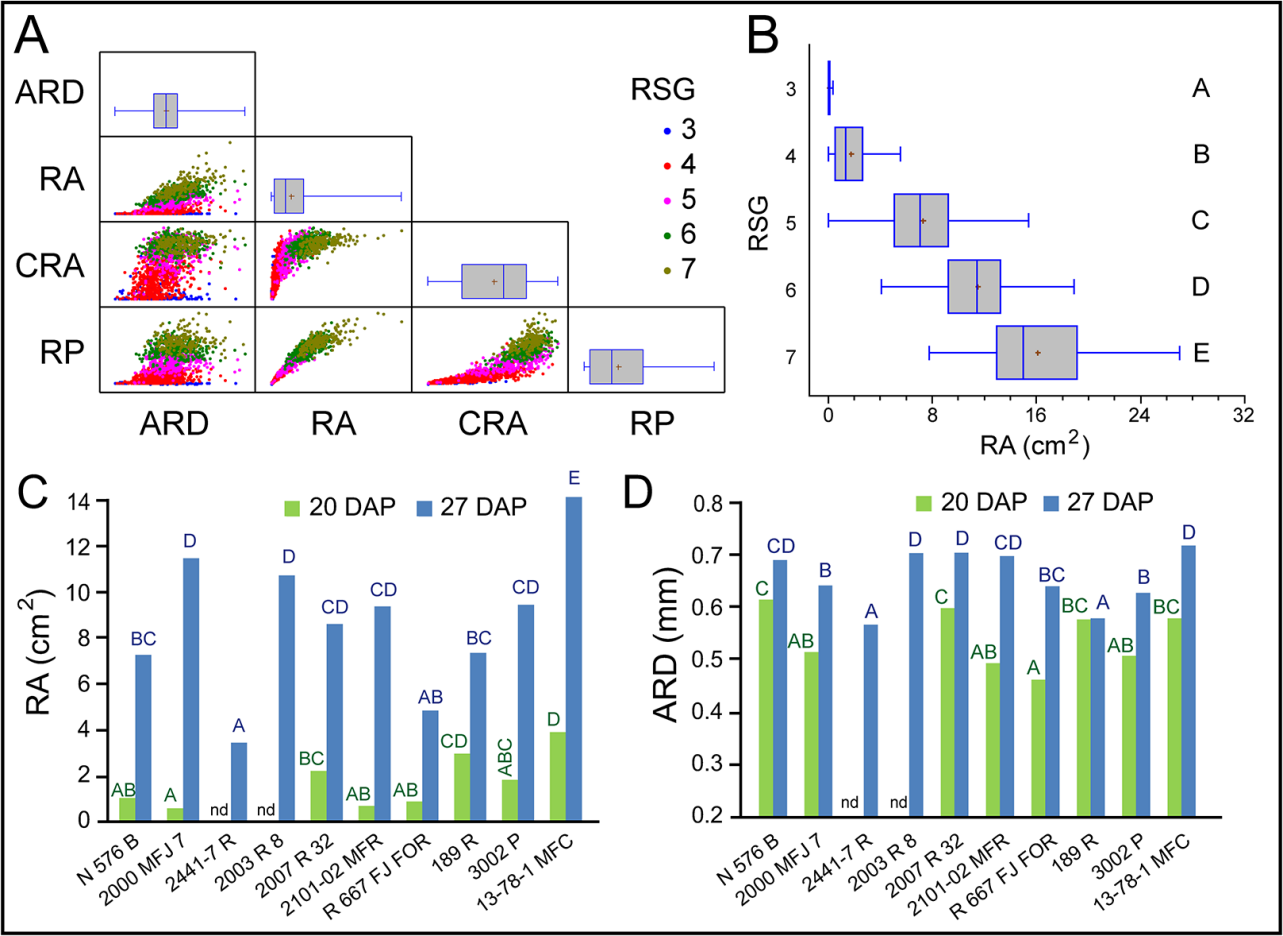
Quantitative description of adventitious rooting in carnation stem cuttings grown in soil plugs. (A) Scatter plots of some root system parameters as defined in Table 2. Data are color-clustered based on their RSG values. Box-plots of each parameter are also represented. (B) Box-plots of RA according to RSG values. Different letters indicate significant differences (*P* < 0.01) over RSG values. Graphic representation of average (C) RA and (D) ARD values in different carnation cultivars over time. Different letters indicate significant differences (*P* < 0.01) over cultivars for a given time. Nd: not determined.

In addition, some of the root system parameters measured were used for the quantification of adventitious rooting. RSG and ARD were estimated as the average of the data from the four soil plug images taken for each stem cutting sample. Whereas RA, CRA and RP were the sum of the data measured from the four soil plug images of each stem cutting sample. We found a positive and highly significant correlation between RA and RP, as well as between RSG and either RA or RP (Fig 6A and S2 Table). Root system area, estimated by RA, displayed a 10-fold variation ranging from an average value of 1.54 ± 1.23 cm^2^ in stem cuttings scored as RSG = 4 to 14.95 ± 3.75 cm^2^ in stem cuttings scored as RSG = 7 (Fig 6B). Similar analyses were performed for RP and ARD (S3A and S3B Fig). Multifactorial ANOVA tests for each parameter as regards cultivar type, rooting performance, batch experiment and DAP were performed as described above. RGS, RA and RP were significantly influenced by DAP and by cultivar type. ARD was mainly dependent on DAP. A residual batch effect was also found for each of these parameters. Unexpectedly, the rooting performance of the studied cultivars during their commercial production was not associated with the observed differences in adventitious rooting in our experiment, which could be explained by the lack of the exogenous auxin treatment in the later.

RP, RA and CRA values differed significantly in all cultivars between 20 and 27 DAP (Fig 6C and S3C and S3D Figs), with the exception of those cultivars without rooting data at 20 DAP (*2441–7 R* and *2003 R 8*). RA was used here as an estimator or rooting performance. At 20 DAP, the studied cultivars clustered in four groups (Fig 6C), with average RA values ranging from 0.56 ± 0.55 cm^2^ in *2000 MFJ 7* to 3.85 ± 2.52 cm^2^ in *13-78-1 MFC*. One week later, five groups were observed (Fig 6C and Table 3). Average RA values at 27 DAP ranged between 3.39 ± 3.05 cm^2^ in *2441–7 R* and 15.32 ± 4.53 cm^2^ in *13-78-1 MFC*. Similar results were found for RP values (S3C Fig). Root diameter was estimated by mean ARD data and ranged between 0.49 ± 0.10 mm in *2101–02 MFR* at 20 DAP and 0.71 ± 0.10 mm in *3002 P* at 27 DAP (Fig 6D and Table 3). For each cultivar, rooting speed was estimated as the average of the daily increase in RA between 27 and 20 DAP (expressed in cm^2^/day). We found a 4-fold variation in rooting speed, the extremes represented by *2441–7 R* (0.12 ± 0.11 cm^2^/day) and *13-78-1 MFC* (0.42 ± 0.15 cm^2^/day) (Table 3).

**Table 3:** Some parameters describing adventitious rooting in carnation stem cuttings grown in soil plugs.

Cultivar code^a^	RSG	RA (cm^2^)	ARD (mm)	Rooting speed (cm^2^/day)	Rooting performance
13-78-1 MFC	6.47 ± 0.57 F	15.32 ± 4.53 E	0.72 ± 0.13 D	0.42 ± 0.15 E	Good
189 R	5.50 ± 0.81 DE	7.27 ± 3.29 BC	0.58 ± 0.09 A	0.17 ± 0.07 AB	Poor
2000 MFJ 7	5.89 ± 0.45 EF	11.36 ± 3.03D	0.64 ± 0.10 B	0.39 ± 0.10 DE	Good
2003 R 8	5.43 ± 0.66 CDE	10.62 ± 3.83 D	0.70 ± 0.09 D	0.38 ± 0.14 DE	Intermediate
2007 R 32	4.88 ± 0.94 BC	8.52 ± 5.83 CD	0.70 ± 0.15 D	0.23.± 0.14 BC	Intermediate
2101–02 MFR	5.85 ± 1.22 EF	9.28 ± 4.91 CD	0.69 ± 0.11 CD	0.33 ± 0.16 CD	Intermediate
2441–7 R	4.14 ± 0.65 A	3.39 ± 3.05 A	0.56 ± 0.11 A	0.12 ± 0.11 A	Bad
3002 P	5.51 ± 1.01 CDE	9.35 ± 4.79 CD	0.64 ± 0.13 B	0.23 ± 0.14 BC	Poor
N 576 B	4.95 ± 1.00 BCD	7.18 ± 4.63 BC	0.69 ± 0.13 CD	0.25 ± 0.16 BC	Poor
R 667 FJ FOR	4.66 ± 0.98 AB	4.78 ± 4.25 AB	0.65 ± 0.13 BC	0.14 ± 0.11 A	Bad

^a^A minimum of 25 stem cuttings were analyzed, except for *2441–7 R* (n = 16). Average RSG, RA and ARD correspond to those measured at 27 DAP. Different letters indicate significant differences (*P* < 0.01) between the cultivars.

Taken together, we categorized the studied cultivars regarding rooting performance in four groups (Table 3): (i) bad-rooting cultivars (*2441–7 R* and *R 667 FJ FOR*), (ii) poor-rooting cultivars (*N 576 B*, *189 R* and *3002 P*), (iii) intermediate-rooting cultivars (*2003 R 8*, *2007 R 32* and *2101–02 MFR*), and (iv) good-rooting cultivars (*2000 MFJ 7*, and *13-78-1 MFC*).

### Correlations between morphometric, ecophysiological and adventitious rooting traits in cultivated carnation

We wondered whether we could predict rooting performance based on a given morphometric parameter of the vegetative stem cutting. We found low correlations for some stem cutting and root parameters (S2 Table) that prevented us for root performance assignment based on stem cutting morphology alone. Next, we performed PC analysis on nine parameters: CW, logCP, logCA, CCA, logALS, ARD, RA, CRA and RP (see Materials and Methods). Three PCs accounted for 88.2% of the variation among the studied samples. PC1 explained 56.2% of the total variance. PC2 and PC3 accounted for 18.6 and 13.4% of the variance, respectively (S3A Table). Consistently with the low correlation found between vegetative and rooting parameters, PC2 clearly separates vegetative and rooting traits, while PC3 is mainly influenced by leaf width, estimated by ALS (S4A Fig and S3B Table). These results are consistent with the good-rooting behavior observed for spray cultivars (S4B Fig), which otherwise display smaller stem cutting size than those of standard cultivars (S4C Fig). Interestingly, PC1 was positively dependent on overall size parameters of both the vegetative and the rooting part of the stem cutting, suggesting that, at least in part, the rooting performance is positively influenced by vegetative stem cutting size.

Also, a limited number of significant correlations were found between rooting parameters and ecophysiological traits (S2 Table). A highly significant and negative correlation was found between RA (or RSG) and CWC, while rooting speed was positively correlated with those variables measuring water and dry matter content ratio, such as LDMC (*r* ^2^ = 0.65). In addition, two PCs accounted for 85.3% of the variation among the studied samples regarding rooting parameters and ecophysiological traits (S5A Fig). The cultivars studied were nicely scattered along these two PCs as it was shown in the factor map diagram (S5B Fig).

### Time course experiment of adventitious rooting in cultivated carnation

**Fig 7:**
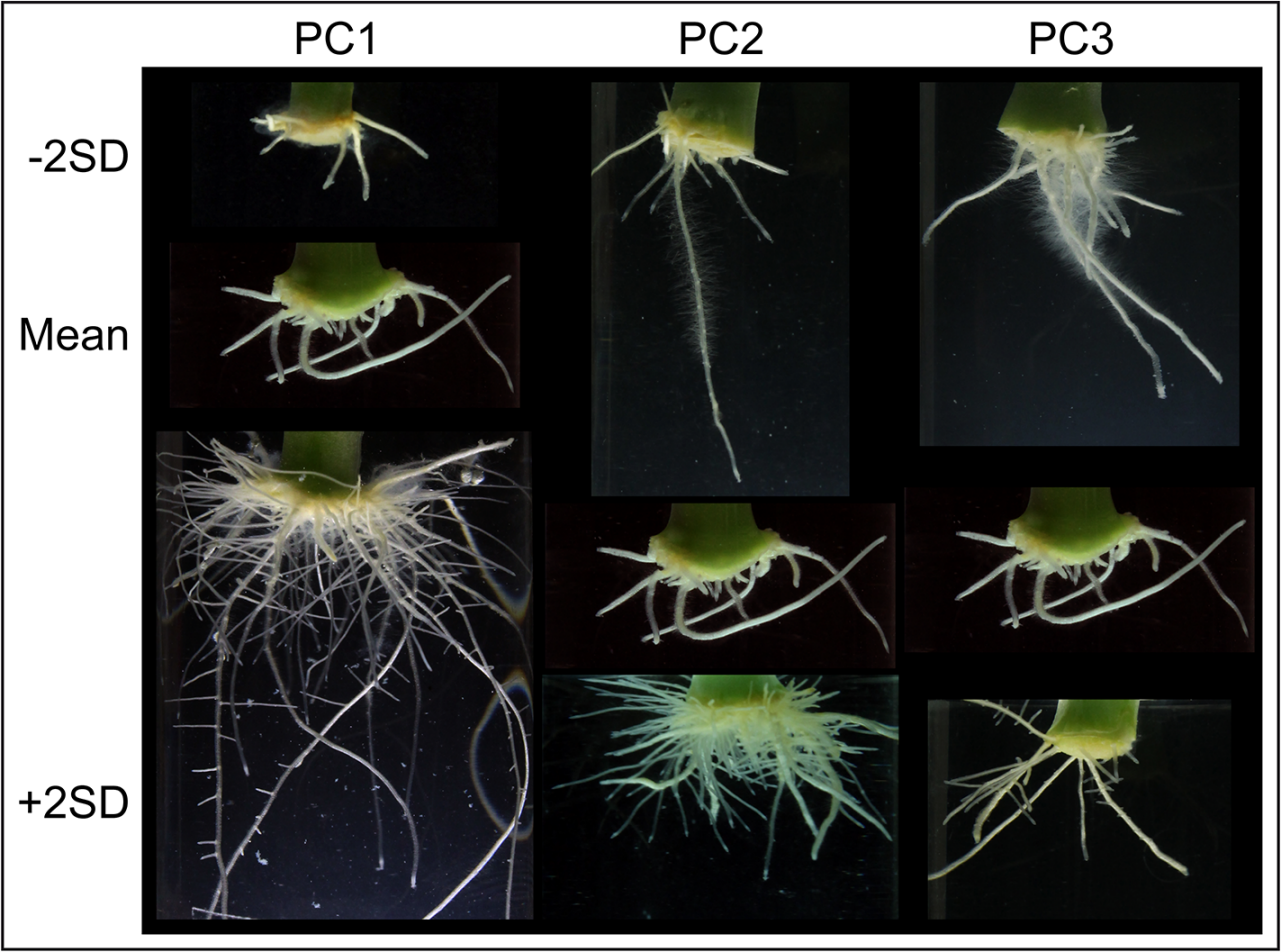
Variation in root system architecture among carnation stem cuttings grown *in vitro*. For each PC, a representative image corresponding to minus or plus two times the standard deviation (−2SD and +2SD) over the mean is shown.

To provide some understanding into the observed differences in adventitious rooting between the studied cultivars, we morphometrically characterized root system architectural traits in the base of the stem cuttings of six of these cultivars between 13 and 29 DAP to environmentally-controlled *in vitro* conditions and without exogenous auxin treatment (see Materials and Methods). For each stem cutting, we gathered quantitative data of 19 previously stablished root system architectural traits [12]. We first reduced the number of studied parameters by partial correlation analysis. Then, iterative PC analysis (see Materials and Methods) allowed us to select for further studies the most relevant parameters, which were related to root network size (RL, RA, RD, RW, ARD and MXR) or root network distribution (RWD, RLD, and RS). Three PCs accounted for 84.7% of the observed variation. PC1 explained 55.3% of the total variance. PC2 and PC3 accounted for 18.1 and 11.3% of the variance, respectively (S4 Table). To visualize the effects of PC1, PC2 and PC3 (S6 Fig) on root architecture, representative images are depicted in Fig 7, where the PC values vary plus or minus two SDs from the mean. PC1 mostly accounted for differences in the size of the root system whereas PC2 and PC3 affected specific attributes of the spatial distribution of the root network.

**Fig 8:**
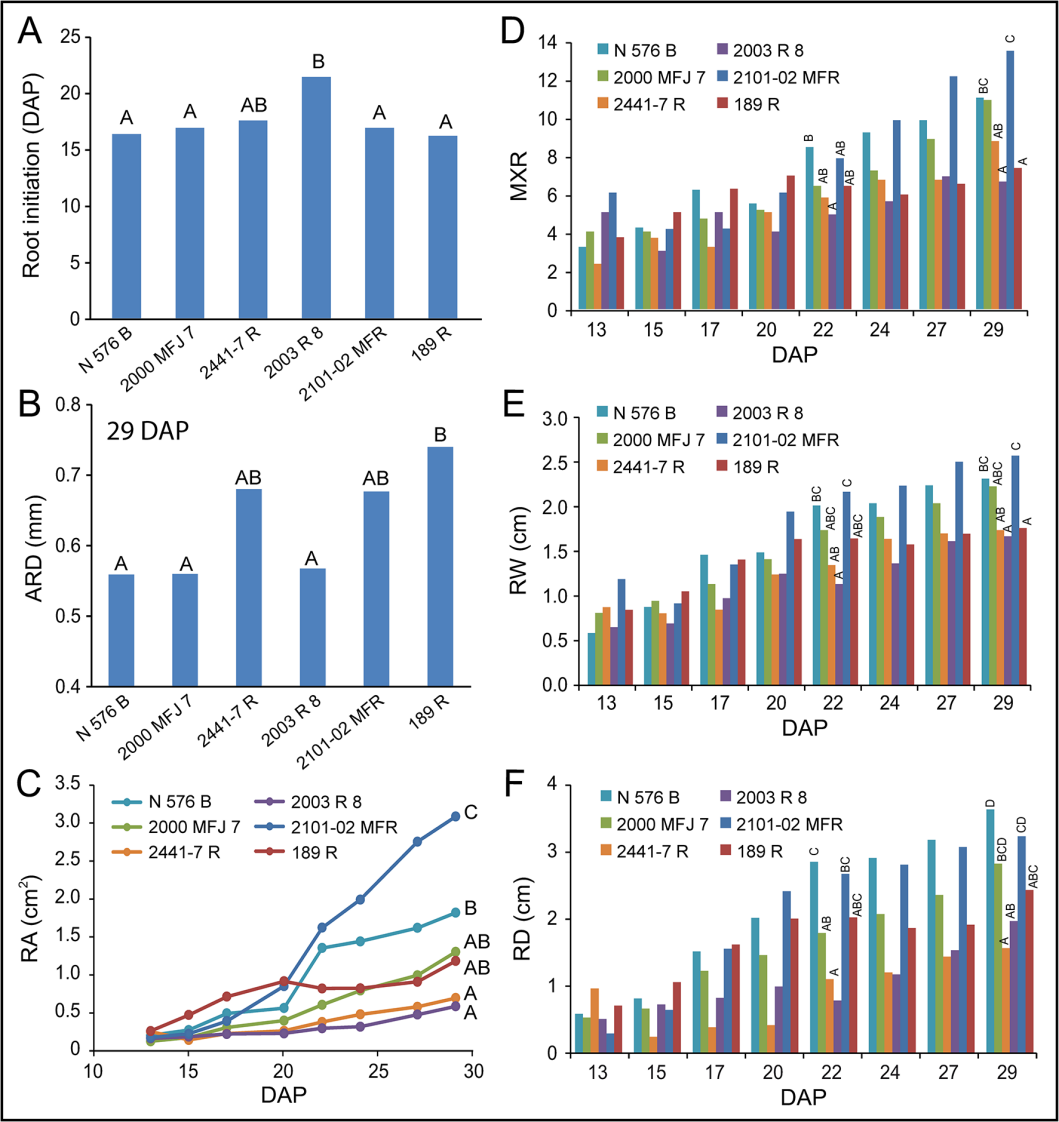
Quantitative description of adventitious rooting in carnation stem cuttings grown *in vitro*. (A) Root initiation and average root diameter (B) values are shown for the studied cultivars. Asterisks indicate significant differences (*P* < 0.05) over time for a given cultivar. (C-F) Time-course analysis of some root network parameters: (C) area, (D) root number, (F) depth, and (F) width. Different letters indicate significant differences (*P* < 0.01) between the cultivars for a given time (13, 22 or 29 DAP).

**Fig 9:**
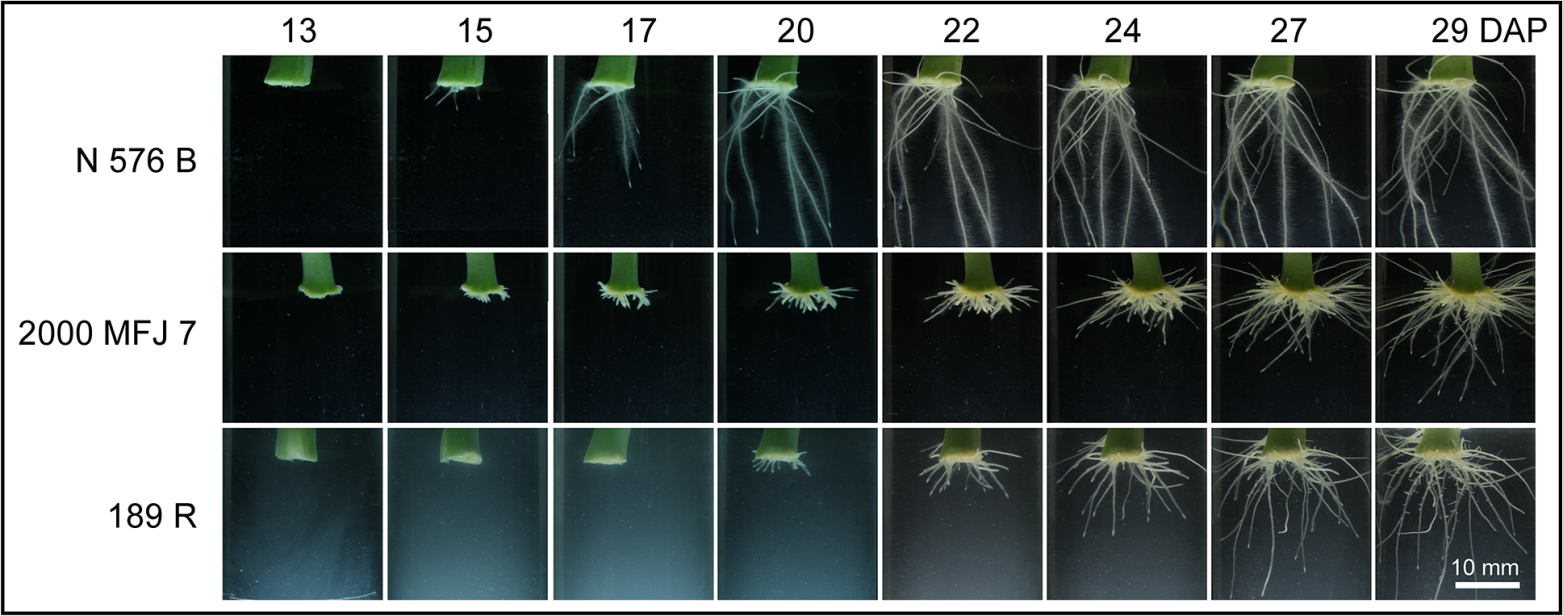
Time-series of adventitious rooting in some carnation cultivars grown *in vitro*. A representative stem cutting was imaged between 13 and 29 DAP for each cultivar.

We found highly significant and positive correlations for most of the parameters studied measuring root network size except ARD (S7A Fig and S5 Table). Multifactorial ANOVA tests indicated that all the studied traits were strongly dependent on cultivar genotype and DAP (data not shown). For each cultivar, we estimated root initiation as the time when the roots were first observed in the stem cutting base. Consistent with our results obtained on soil plugs (see above), the *2003 R 8* and *2441–7 R* cultivars displayed a significant delay in the initiation of adventitious roots compared to that of the other cultivars studied (Fig 8A). Interestingly, ARD values were significantly increased along the experiment in all the studied cultivars and remained low in some cultivars with bad-rooting performance, such as *2441–7 R* (Fig 8B and Table 4). Root growth was estimated by the concomitant increase in RA along the experiment. The differences in RA values between any two cultivars were apparent after 20 DAP (Fig 8C). Similar results were found for the total length of the root network (S7B Fig). In addition, the cultivars differed in the number of emerged roots from the stem cutting base, as estimated by the MXR parameter (Fig 8D). Therefore, the largest root system developed by the *2101–02 MFR* cultivar is likely due to its higher number of roots and faster growth than other spray cultivars, such as *2000 MFJ 7* (Fig 9). On the other hand, standard cultivars such as *2003 R 8*, and *2441–7 R* displayed smaller root systems because of a delay in their root emergence and their slow root growth (data not shown). Another important parameter that characterizes root system architecture is maximum root depth as it is known that deeper rooting improves water and nutrient capture in different environments [18, 19]. In general, spray cultivars display deeper and wider root systems than standard cultivars at 29 DAP (Fig 8E and 8F, and Table 4). However, the *N 567 B* standard cultivar displayed a good-rooting behavior as it produced a higher number of roots and longer than those of other standard cultivars (Fig 8). Three additional parameters (RWD, RLD and RS) accounted for the spatial distribution of the root network. RWD and RLD values were relatively constant for most of the studied genotypes along the experiment (S7C and S7D Fig). In contrast, RS values decreased during rooting, which reflected the transition between a compact root system at 15 DAP to a wider and less dense root system at 29 DAP (Fig 9 and S7E Fig).

**Table 4:** Some parameters describing adventitious rooting in carnation stem cuttings grown *in vitro*.

Cultivar code^a^	RA (cm^2^)	ARD (mm)	MXR	RD (mm)	Rooting performance
189 R	1.22 ± 1.01AB	0.76 ± 0.18 B	7.27 ± 3.83 A	2.43 ± 1.29 ABC	Intermediate
2000 MFJ 7	1.34 ± 0.76 AB	0.57 ± 0.18 A	10.77 ± 3.94 BC	2.83 ± 1.33 BCD	Intermediate
2003 R 8	0.61 ± 0.40 A	0.57 ± 0.19 A	6.57 ± 2.74 A	1.97 ± 1.19 AB	Bad
2101–02 MFR	3.16 ± 1.68 C	0.69 ± 0.16 AB	13.32 ± 4.98 C	3.16 ± 1.68 CD	Good
2441–7 R	0.72 ± 0.52 A	0.69 ± 0.25 AB	8.67 ± 3.94 AB	1.57 ± 1.15 A	Bad
N 576 B	1.87 ± 0.92 B	0.57 ± 0.16 A	10.90 ± 3.78 BC	3.64 ± 0.94 D	Good

^a^A minimum of 15 stem cuttings were analyzed, except for *2441–7 R* (n = 5). Average RA, ARD, MXR and RD correspond to those measured at 29 DAP. Different letters indicate significant differences (*P* < 0.01) between the cultivars.

In our *in vitro* rooting experiment, two groups of cultivars displayed contrasting rooting-performance. On the one hand, the *2101–02 MFR* spray cultivar displayed a good-rooting behavior. On the other hand, the *2003 R 8* and *2441–7 R* standard cultivars showed a bad-rooting behavior. Interestingly, one standard cultivar (*N 576 B*) and one spray cultivar (*2000 MFJ 7*) displayed similar rooting behavior which was achieved by potential different mechanisms (Fig 9).

## Discussion

The wide variation range of stem cutting losses observed during commercial production at the Barberet & Blanc’s rooting station was strongly dependent on the cultivar type. As such, most of the cultivars with high stem cutting losses (>5%) were of the standard type, while those with the lowest stem cutting losses were of the spray type. Besides, only 12 cultivars displayed stem cutting losses larger than 7%, a result which is consistent with this trait being under strong negative selection during the development of new cultivars.

Stem cuttings are periodically pinched from adult mother plants by skilled operators based on their external attributes, named size, color, leaf number and morphology, and are kept cold for long-term storage. For the studied cultivars in this work, our results are consistent with the fact that stem cuttings in spray cultivars are usually smaller than those of standard cultivars, irrespectively of the environmental variables, suggesting that these differences are genetically encoded. Four morphological parameters of the stem cuttings (CP, ALS, LN and the CCA/CA ratio) were shown to be informative for cultivar discrimination and the differences in CP between any two given time points allowed us to measure stem cutting growth during rooting. Stem cutting growth was mainly restricted to the younger leaves [2], while the changes in older leaves during rooting were limited to their insertion angle and more folding of their lamina along the midrib. As a diversity of crop plants has been observed to alter leaf angle in response to water deficit [20], the reported changes in leaf angle could be caused by the dehydration of older leaves occurring during the early steps of rooting.

We observed statistically-significant differences amongst some ecophysiological traits for the studied cultivars (Table 1). It’s been shown previously that high SLA implies a greater surface to volume ratio in leaves and therefore a higher water loss, whereas high photosynthetic rates is linked to high transpiration and lower drought tolerance [21]. The studied carnation cultivars displayed lower SLA and a slightly higher GS values than those of their close relative from temperate regions, *D*. *barbatus*, suggesting adaptation to a Mediterranean habitat characterized by high-light and dry climate.

For commercial production of rooted cuttings at the breeders’ rooting station, cold-stored cuttings are usually treated with exogenous auxin prior to planting them to soil plugs. To better describe the differences in adventitious rooting that are caused by endogenous (genetic) factors, we planted freshly-harvested cuttings from ten cultivars differing in their commercial rooting losses directly on soil plugs without the addition of exogenous auxin. As expected, the studied cultivars showed remarkable differences in quantitative rooting parameters such as total root area (RA) between cultivars and at different times after planting. Rooting speed, estimated by the observed change in RA, was one of the best indicators of the rooting performance in the studied population. Interestingly, the average root diameter (ARD) was found negatively correlated with rooting performance, and the poor-rooting cultivars always displayed thinner roots than the good-rooting cultivars. To support a quick assignment of rooting performance for the new carnation cultivars being bred, we defined a qualitative scale based on seven non-overlapping rooting stages (RSG; Fig 5A). We found a strong and positive correlation between some root system size parameters (RA, RP) and RSG, which validated RSG for the fast determination of rooting performance *in situ*. A trained person is able to visually assign RSG to soil plug images at a rate of about 800 images per hour. Thus, the RSG parameter could be easily scored on the same stem cutting at different time-points after planting. To increase discrimination power of this parameter, intermediate rooting stages could be added.

Based on the data obtained from stem cuttings grown in soil plugs, we assigned the ten studied cultivars to four groups that differed in their rooting performance from bad-rooting cultivars to good-rooting cultivars. Curiously, *R 667 FJ FOR* was initially selected because of its low rooting losses during commercial production but it behaved as a bad-rooting cultivar when cold-storage and exogenous auxin treatment were not applied.

From our PC analysis on the variation found in stem cutting morphology (shoot and root traits), we concluded that two different mechanisms could account for the observed differences. On the one hand, PC1 values were dependent on the size of both the shoot region and of the root region, suggesting that common genes are contributing to both traits. On the other hand, PC2 clearly separates vegetative and root traits. Consistent with our results, a recent study in wheat using a double-haploid mapping population found a significant and positive correlation between plant height and some components of root architecture [22]. Indeed, a few QTLs for root and shoot traits were found co-localized in this work. This indicates that there are common genes, or at least closely linked genes, contributing to both traits. Consistent with our results too, other genes only influence root traits or plant height [22].

Many studies have been carried out to highlight the relationship between leaf and root parameters during drought adaptation [23, 24] but not much is known about the association between leaf and root traits in relation to adventitious rooting. On the one hand, we found that rooting performance in carnation cuttings was negatively correlated with their water content, suggesting that a drought stress signal might induce AR formation in these species. On the other hand, rooting speed was found positively correlated with the water/dry weight ratio, so cultivars accumulating higher (sugar) resources might be able to surpass the restriction from water excess over AR formation. Interestingly, the studied cultivars were perfectly separated by only two PCs including a small number of rooting and ecophysiological parameters (S5 Fig). Further experiments are required to narrow down this observation and to shed some light into the complex link between water stress, nutrient content and AR formation.

In addition, we performed a time-course analysis of adventitious rooting in carnation stem cuttings by following a novel *in vitro* approach using transparent agar tubes in a controlled environment. Similar approaches have been used for the study of root traits in other crops, such as maize [25] or rice [26, 27]. Quantitative data describing the root system architecture was obtained for six carnation cultivars from 13 to 29 DAP using a previously stablished root analysis software [12]. Although this software was initially designed for the analysis of primary and lateral roots, we demonstrated its application for the quantitative analysis of ARs. Three PCs including nine parameters accounted for most of the variation found in adventitious rooting, which was mainly triggered by differences in the overall size of the root system (Fig 7). Among the parameters measured, total root length and root depth are quite significant as they determine capture of water and nutrients by plants, and are targets for crop improvement [28]. Indeed, Kirkegaard *et al*. (2007) demonstrated that a relatively small increase in rooting depth in wheat could provide a significant yield increase in this species [29]. Consistent with these results, the good-rooting carnation cultivars *N 576 B* (standard) and *2010–02 MFR* (spray) displayed the deeper root system among the studied ones. The latter cultivar also showed higher number of roots and faster growth than other spray cultivars. Standard cultivars, such as *2003 R 8*, displayed smaller root systems mainly due to a delay in root emergence and slow root growth. Interestingly, the bad rooting behavior of *2003 R 8* observed in our experiments was partly rescued by exogenous auxin application [30], which might explain the differences in rooting performance during commercial production.

By the detailed study of the time-series of adventitious rooting in several carnation cultivars described in this work, we derived general principles that might account for the differences in rooting performance observed between other carnation cultivars. The bad-rooting cultivars are characterized by one or several of the following attributes: (i) a delay in root initiation from the stem cutting base, (ii) a reduced number of AR primordia, (iii) a slow elongation rate of primary ARs, and (iv) slow initiation and elongation rates of secondary ARs. A novel semi-automated image analysis platform is being implemented in our lab (S. Tormos-Moltó and J.M. Pérez-Pérez, unpublished) which will allow the continuous monitoring of AR growth from carnation stem cuttings over a long-term period (>1 month). Using this platform, we will interrogate the extensive germplasm collection at Barberet & Blanc for carnation cultivars displaying specific alterations in their AR system.

To get some insight into the genetic influence in shoot and/or root traits in carnation stem cuttings, a similar approach to that described elsewhere [22], needs to be undertaken. To this end, two carnation cultivars extensively differing in both their stem cutting morphology and their rooting performance, and for which some genetic information is already known, will need to be crossed and their descendants studied at the genetic and the phenotypic levels. The molecular signature of the different stages of adventitious rooting in the two contrasting cultivars *2101–02 MFR* and *2003 R 8* that have been studied in this work will be presented elsewhere [30], which makes them appropriate parents of the mapping population required for QTL analysis. In addition, a combined approach for non-targeted metabolite and hormone profiling in carnation cultivars displaying contrasting AR formation responses will shed some light in the biochemical signatures of this process. By these additional approaches that have been initiated in our lab, we intend to contribute to the basic understanding of the molecular events leading to the complex developmental response of AR formation which will help to establish a marker-assisted selection approach to select for improved adventitious rooting in this and other ornamental species.

## Supporting Information

S1 Fig Distribution of average rooting losses among carnation cultivars. The data from the studied population (n = 132 cultivars) fit the log-normal distribution. Values indicate average ± SD (in %).
(TIF)

S2 Fig Graphic representation of some stem cutting parameters in different carnation cultivars over time. Asterisks indicate significant differences (*P* < 0.05) over time for a given cultivar. Nd: not determined.
(TIF)

S3 Fig Graphic representation of some rooting parameters in different carnation cultivars grown in soil plugs. (A-B) Box-plots of RP (A) and ARD (B) data according to RSG values. Different letters indicate significant differences (*P* < 0.01) between RSG values. (C-D) Graphic representation of average RP (C) and CRA (D) values in different carnation cultivars over time. Different letters indicate significant differences (*P* < 0.01) between the cultivars for a given time. Nd: not determined.
(TIF)

S4 Fig Principal component analysis of stem cutting morphology and adventitious rooting parameters. (A) Graphical representation of PC1 and PC2. (B-C) Representative images of two good-rooting stem cuttings of a spray cultivar (B) and a standard cultivar (C) displaying contrasting stem cutting morphology and similar rooting performance. Images were obtained as described in Materials and Methods at 27 DAP.
(TIF)

S5 Fig Principal component analysis of stem cutting ecophysiology and adventitious rooting parameters. (A) Principal component analysis of rooting and ecophysiological parameters. Graphical representation of PC1 and PC2 are shown. (B) Individual factor map for PC analysis of rooting and ecophysiological parameters. Cultivars have been color-coded as regards their rooting performance (red: bad; orange: poor; blue: intermediate; green: good).
(TIF)

S6 Fig Principal component analysis of adventitious rooting parameters measured in carnation stem cuttings grown *in vitro*. Representation of PC1, PC2 and PC3 in a tridimensional space. Root system parameters are represented as defined in Table 2.
(TIF)

S7 Fig Quantitative description of adventitious rooting in carnation stem cuttings grown *in vitro*. (A) Scatter plots of some adventitious rooting parameters as defined in Table 2. Box-plots of each parameter are also represented. Graphic representation of RL (B), RLD (C), RWD (D) and RS (E) values in different carnation cultivars over time. Different letters indicate significant differences (*P* < 0.01) between the cultivars for a given time.
(TIF)

S1 Table Carnation cultivars studied in this work. (PDF)

S2 Table Linear correlation matrix of average stem cutting parameters measured. (PDF)

S3 Table Principal component analysis of root system parameters in carnation stem cuttings grown in soil plugs. (PDF)

S4 Table Principal component analysis of root system parameters in carnation stem cuttings grown *in vitro*. (PDF)

S5 Table Linear correlation matrix of root system parameters measuredin carnation stem cuttings grown *in vitro*. (PDF)
