# Supplementary figures and images for: Quantitative Analysis of Adventitious Root Growth Phenotypes in Carnation Stem Cuttings
Source: PLoS One. 2015 Jul 31;10(7):e0133123. doi: 10.1371/journal.pone.0133123 (PMC4521831; doi:10.1371/journal.pone.0133123)

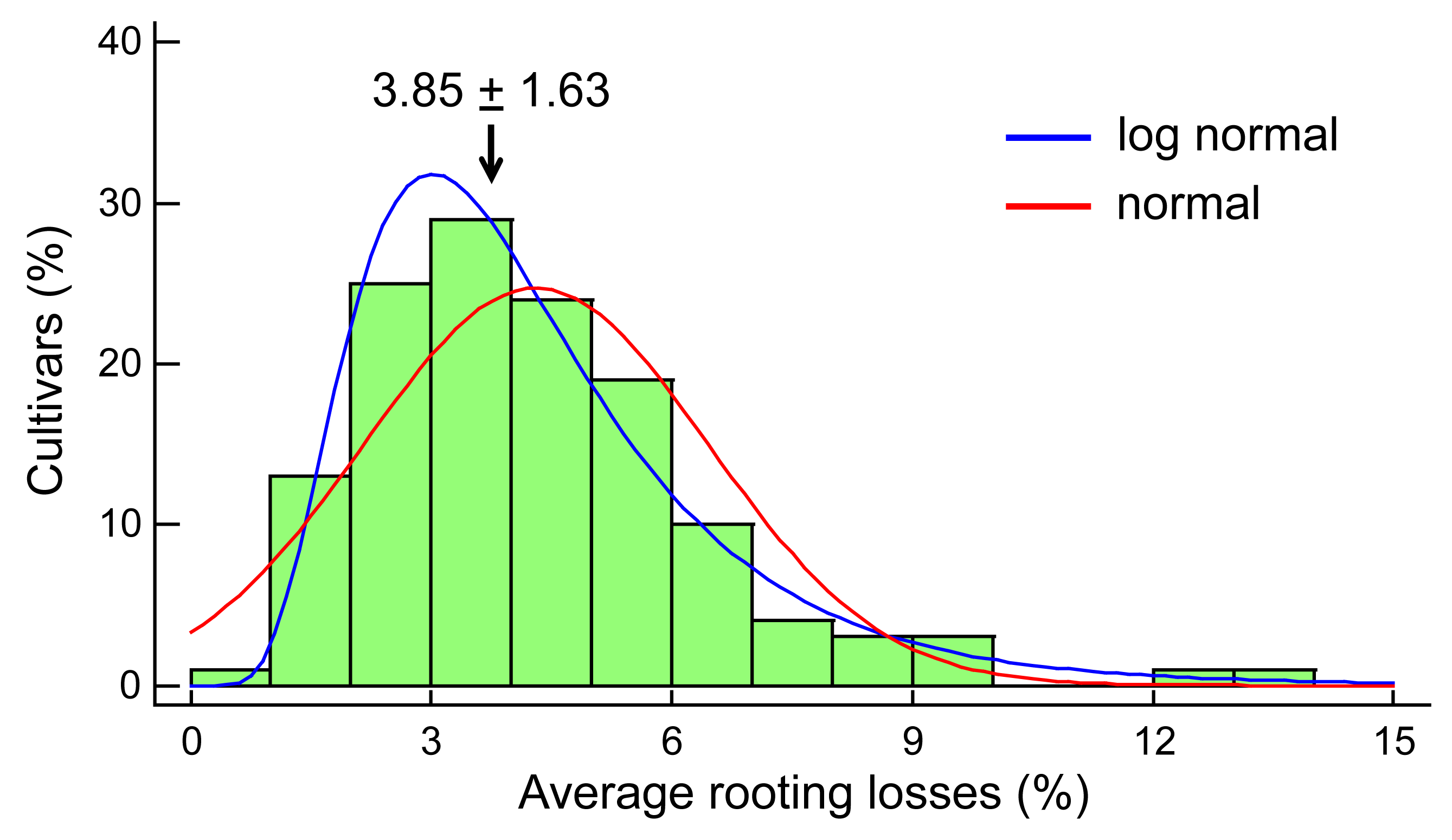

Supplement: S1 Fig — The data from the studied population (n = 132 cultivars) fit the log-normal distribution. Values indicate average ± SD (in %). (TIF) [file pone.0133123.s001.tif]

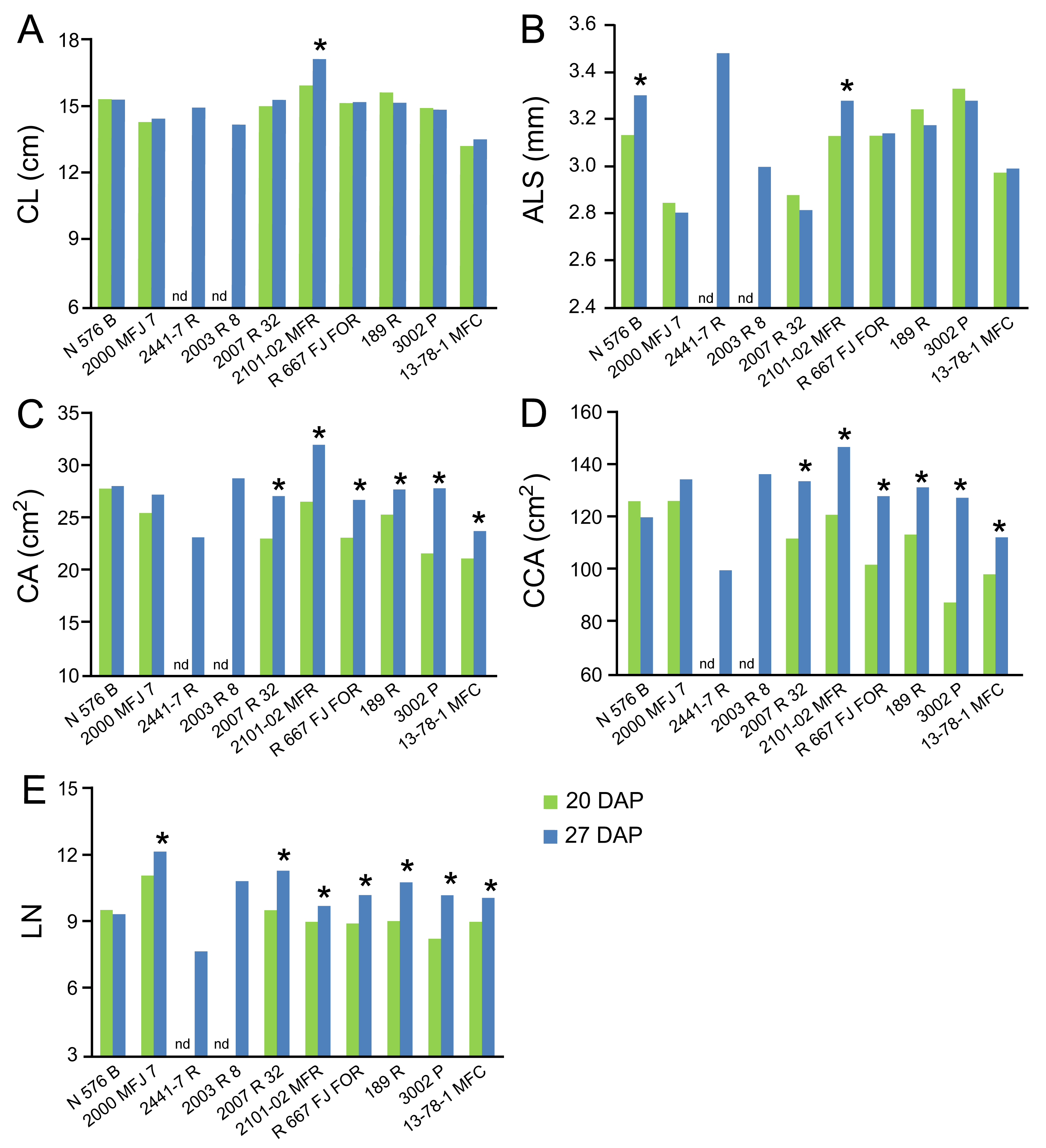

Supplement: S2 Fig — Asterisks indicate significant differences (P < 0.05) over time for a given cultivar. Nd: not determined. (TIF) [file pone.0133123.s002.tif]

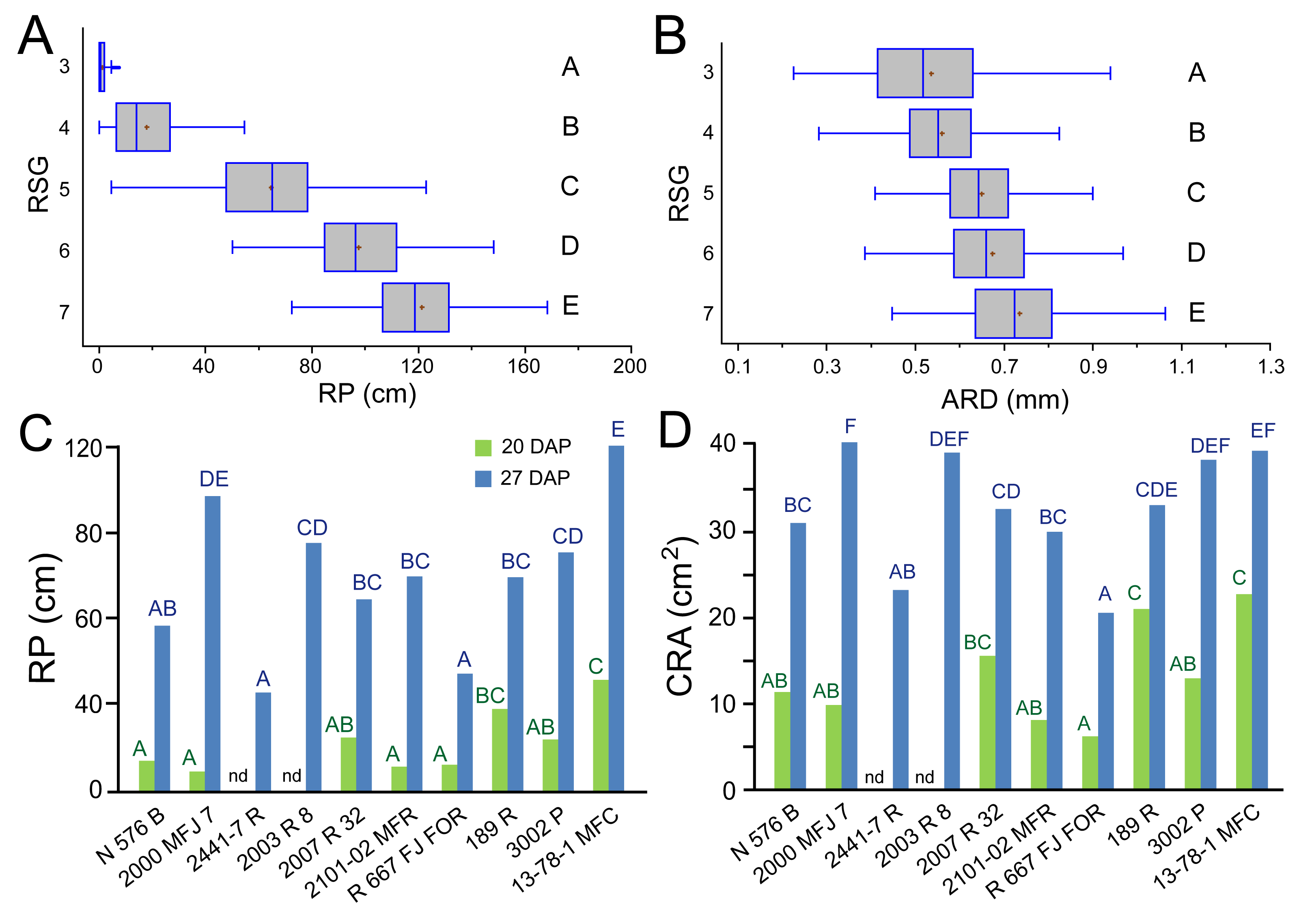

Supplement: S3 Fig — (A-B) Box-plots of RP (A) and ARD (B) data according to RSG values. Different letters indicate significant differences (P < 0.01) between RSG values. (C-D) Graphic representation of average RP (C) and CRA (D) values in different carnation cultivars over time. Different letters indicate significant differences (P < 0.01) between the cultivars for a given time. Nd: not determined. (TIF) [file pone.0133123.s003.tif]

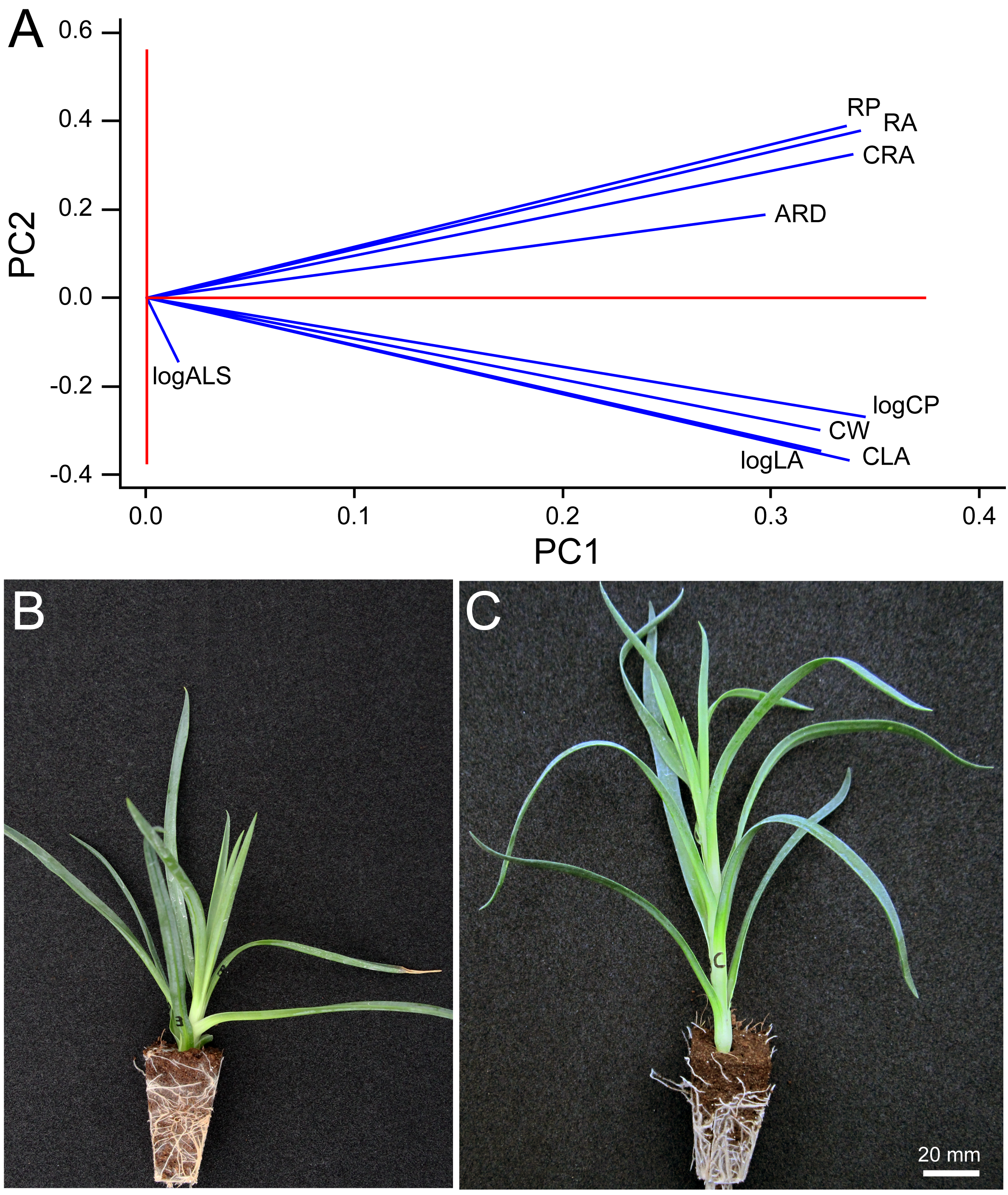

Supplement: S4 Fig — (A) Graphical representation of PC1 and PC2. (B-C) Representative images of two good-rooting stem cuttings of a spray cultivar (B) and a standard cultivar (C) displaying contrasting stem cutting morphology and similar rooting performance. Images were obtained as described in Materials and Methods at 27 DAP. (TIF) [file pone.0133123.s004.tif]

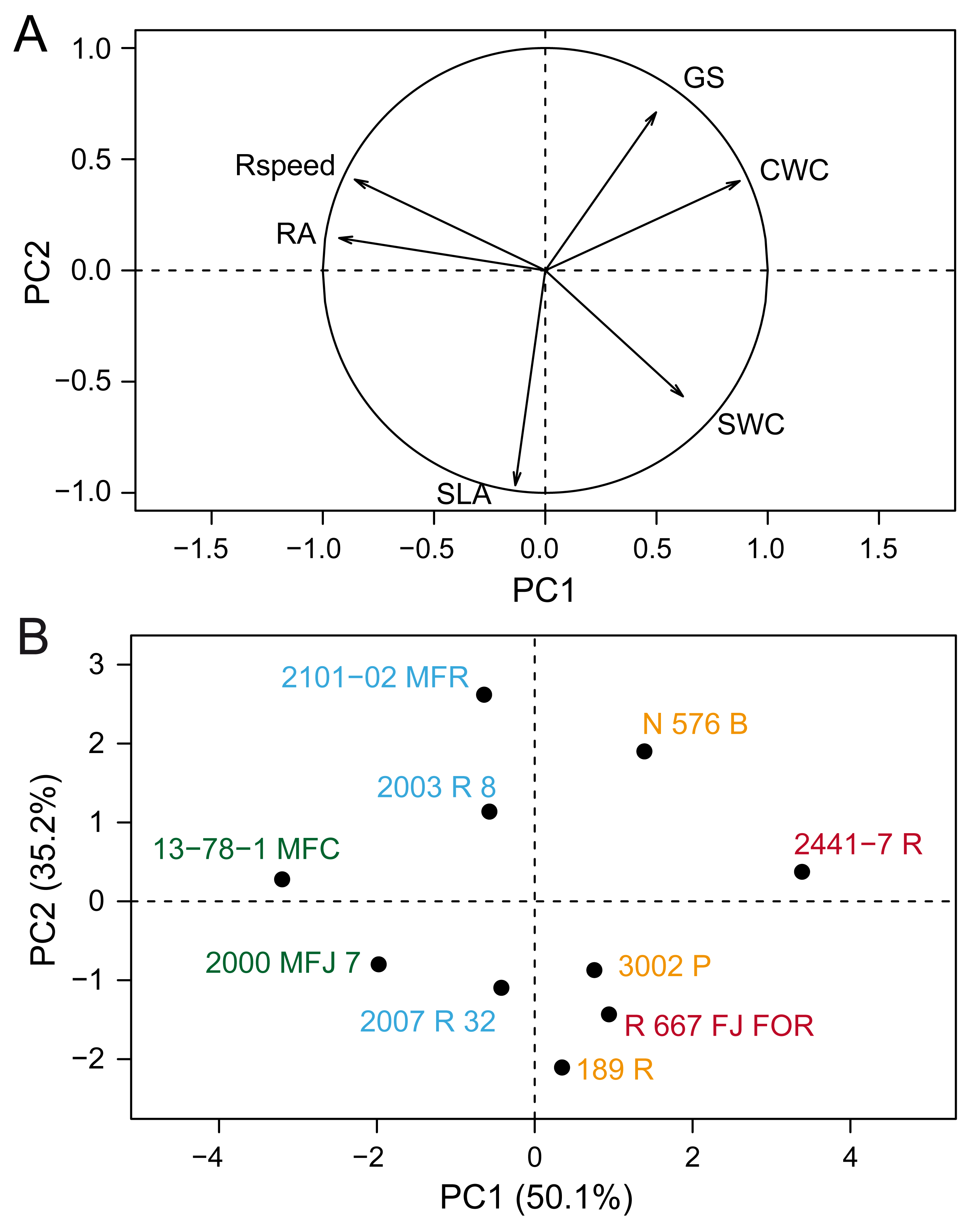

Supplement: S5 Fig — (A) Principal component analysis of rooting and ecophysiological parameters. Graphical representation of PC1 and PC2 are shown. (B) Individual factor map for PC analysis of rooting and ecophysiological parameters. Cultivars have been color-coded as regards their rooting performance (red: bad; orange: poor; blue: intermediate; green: good). (TIF) [file pone.0133123.s005.tif]

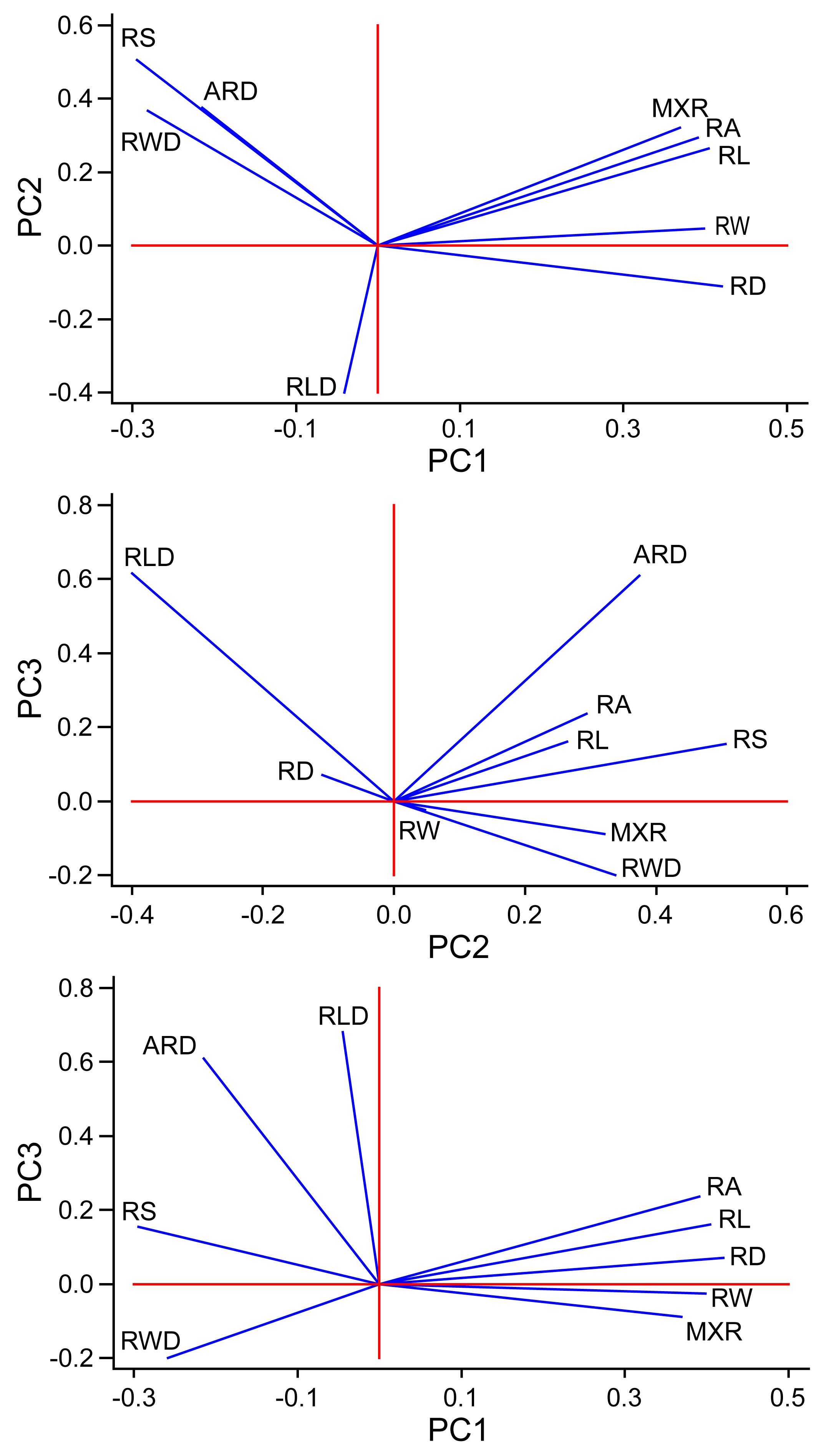

Supplement: S6 Fig — Representation of PC1, PC2 and PC3 in a tridimensional space. Root system parameters are represented as defined in Table 2. (TIF) [file pone.0133123.s006.tif]

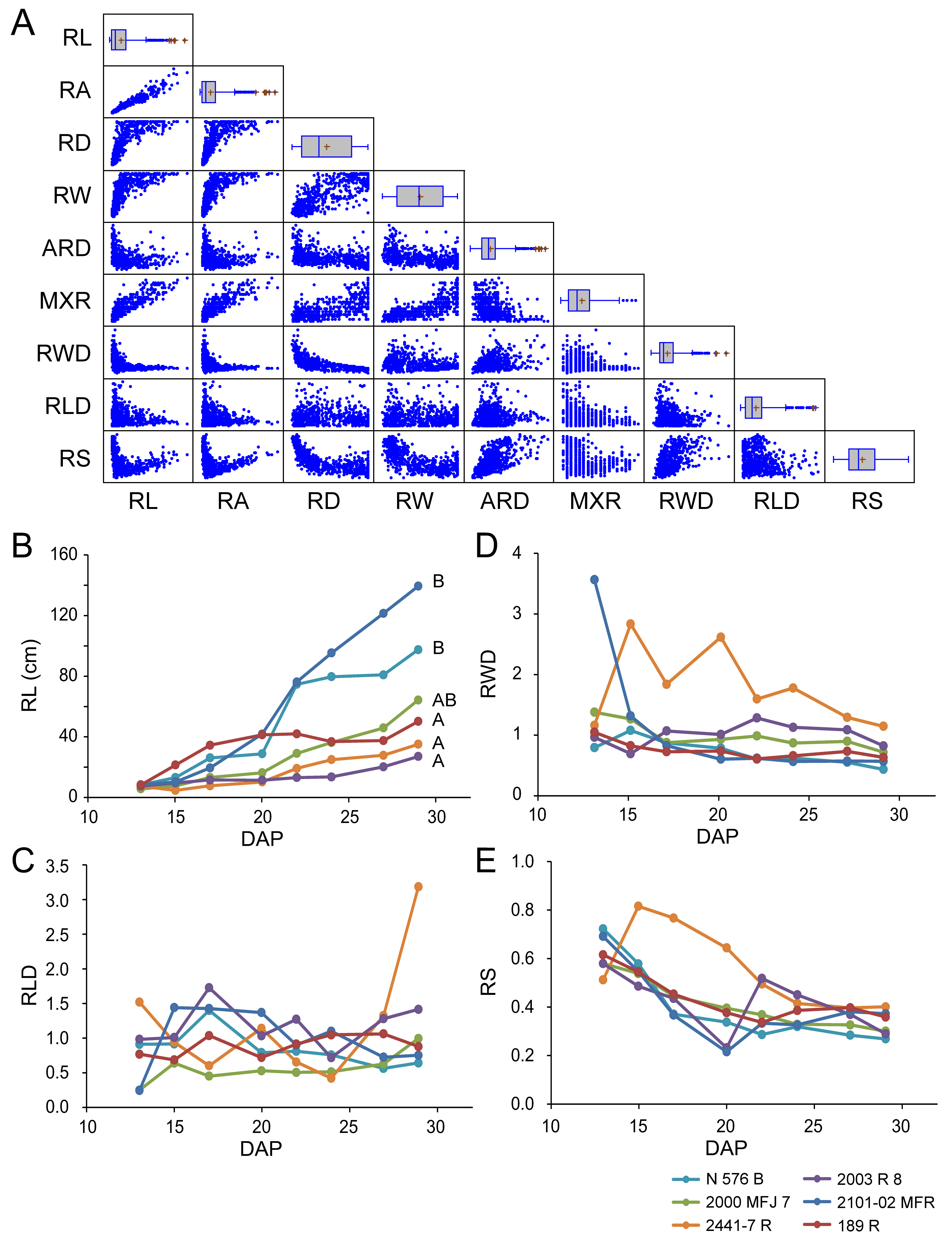

Supplement: S7 Fig — (A) Scatter plots of some adventitious rooting parameters as defined in Table 2. Box-plots of each parameter are also represented. Graphic representation of RL (B), RLD (C), RWD (D) and RS (E) values in different carnation cultivars over time. Different letters indicate significant differences (P < 0.01) between the cultivars for a given time. (TIF) [file pone.0133123.s007.tif]
