# Supplementary material for: Quantitative Analysis of Adventitious Root Growth Phenotypes in Carnation Stem Cuttings
Source: PLoS One. 2015 Jul 31;10(7):e0133123. doi: 10.1371/journal.pone.0133123 (PMC4521831; doi:10.1371/journal.pone.0133123)
Supplement: S1 Table — (PDF) [file pone.0133123.s008.pdf]

Table S1.- Carnation cultivars studied in this work

Cultivar code	Cultivar type	Average rooting losses (%)[a]	Commercial rooting performance[a]	Batch	*In vitro* tested
13-78-1 MFC	Spray	0.83 ± 0.89	Good-rooting	1	No
189 R	Standard	2.60 ± 1.73	Good-rooting	1	Yes
2000 MFJ 7	Spray	3.11 ± 0.80	Intermediate	2	Yes
2003 R 8	Standard	7.29 ± 2.46	Bad-rooting	2	Yes
2007 R 32	Standard	9.03 ± 5.21	Bad-rooting	1	No
2101-02 MFR	Spray	2.02 ± 1.18	Good-rooting	2	Yes
2441-7 R	Standard	8.59 ± 1.11	Bad-rooting	2	Yes
3002 P	Standard	6.29 ± 0.39	Bad-rooting	1	No
N 576 B	Standard	3.78 ± 1.17	Intermediate	2	Yes
R 667 FJ FOR	Standard	1.85 ± 0.71	Good-rooting	1	No

[a]According to empirical data obtained at the breeders' rooting station before the present study.
