# Supplementary material for: Quantitative Analysis of Adventitious Root Growth Phenotypes in Carnation Stem Cuttings
Source: PLoS One. 2015 Jul 31;10(7):e0133123. doi: 10.1371/journal.pone.0133123 (PMC4521831; doi:10.1371/journal.pone.0133123)
Supplement: S2 Table — (PDF) [file pone.0133123.s009.pdf]

Table S2.- Linear correlation matrix of average stem cutting parameters measured

	CW	ALS	CA	CCA	CP	CS	CWL	LN	RSG	ARD	RA	CRA	RP
CL	0.268	0.259	0.601	0.564	0.456	*-0.086*	-0.311	*0.003*	*-0.003*	0.146	*-0.005*	*-0.038*	*-0.044*
CW		*-0.009*	0.641	**0.845**	0.729	-0.610	**0.826**	0.412	0.451	0.413	0.418	0.441	0.406
ALS			0.396	0.028	-0.226	0.450	-0.154	-0.311	*-0.033*	*0.087*	*-0.046*	*0.008*	*-0.060*
CA				0.800	0.782	*-0.070*	0.284	0.419	0.397	0.437	0.399	0.442	0.381
CCA					**0.856**	-0.609	0.499	0.480	0.406	0.426	0.405	0.420	0.384
CP						-0.425	0.451	0.643	0.455	0.423	0.463	0.468	0.452
CS							-0.546	-0.305	-0.200	-0.165	-0.184	-0.187	-0.182
CWL								0.404	0.452	0.331	0.422	0.458	0.431
LN									0.433	0.296	0.448	0.493	0.480
RSG										0.559	**0.897**	**0.837**	**0.920**
ARD											0.694	0.595	0.590
RA												**0.865**	**0.973**
CRA													**0.909**

	Ecut	LA	GS	SLA	LDMC	RSG	ARD	RA
CWC	*-0.147*	*0.513*	**0.849**	*-0.501*	*-0.288*	-0.793	-0.343	-0.757
Ecut		*-0.462*	*-0.504*	0.780	*-0.448*	*-0.342*	*-0.176*	*-0.287*
LA			*0.556*	-0.643	*0.262*	*-0.188*	*0.245*	*-0.264*
GS				-0.659	*-0.250*	*-0.235*	*0.211*	*-0.231*
SLA					*-0.560*	*0.013*	*-0.061*	*0.015*
LDMC						*0.504*	*0.125*	*0.495*

Non-significant correlations ($P > 0.05$) are shown in italics. r values larger than 0.81 ($r^2 \sim 0.65$) are indicated in bold.
