# Supplementary material for: Quantitative Analysis of Adventitious Root Growth Phenotypes in Carnation Stem Cuttings
Source: PLoS One. 2015 Jul 31;10(7):e0133123. doi: 10.1371/journal.pone.0133123 (PMC4521831; doi:10.1371/journal.pone.0133123)
Supplement: S3 Table — (PDF) [file pone.0133123.s010.pdf]

Table S3.- Principal component analysis of root system parameters in carnation stem cuttings grown in soil plugs

3A.- Principal component analysis

Principal component	Eigenvalue	Variance
1	5.0565	56.183
2	1.6777	18.641
3	1.2076	13.418
4	0.4855	5.394
5	0.3170	3.522
6	0.1392	1.546
7	0.0978	1.086
8	0.0112	0.125

3B.- Eigenvectors for the three main principal components (PCs)

Parameters	PC1	PC2	PC3
CW	0.345265	-0.319970	-0.131645
logCP	0.368628	-0.287599	-0.297825
logCA	0.345895	-0.370170	0.263357
CCA	0.360629	-0.392486	-0.097157
logALS	0.016021	-0.152633	0.885513
ARD	0.317331	0.201794	0.164968
RA	0.366201	0.406235	0.030007
CRA	0.362496	0.348536	0.053740
RP	0.358813	0.416310	0.006068
