# Supplementary material for: Quantitative Analysis of Adventitious Root Growth Phenotypes in Carnation Stem Cuttings
Source: PLoS One. 2015 Jul 31;10(7):e0133123. doi: 10.1371/journal.pone.0133123 (PMC4521831; doi:10.1371/journal.pone.0133123)
Supplement: S4 Table — (PDF) [file pone.0133123.s011.pdf]

Table S4.- Principal component analysis of root system parameters in carnation stem cuttings grown *in vitro*

4A.- Principal component analysis

Principal component	Eigenvalue	Variance
1	4.9735	55.262
2	1.6308	18.120
3	1.0161	11.290
4	0.6503	7.225
5	0.4266	4.740
6	0.1195	1.328
7	0.0879	0.976
8	0.0700	0.778

4B.- Eigenvectors for the three main principal components (PCs)

Parameters	PC1	PC2	PC3
RL	0.403784	0.263924	0.161014
RA	0.390869	0.293961	0.236238
RD	0.420354	-0.109710	0.071432
RW	0.397987	0.046778	-0.024799
ARD	-0.213881	0.374525	0.609607
MXR	0.368666	0.320827	-0.088581
RWD	-0.280960	0.367182	-0.217851
RLD	-0.044941	-0.442112	0.679758
RS	-0.294060	0.506184	0.153467
