# Supplementary material for: Quantitative Analysis of Adventitious Root Growth Phenotypes in Carnation Stem Cuttings
Source: PLoS One. 2015 Jul 31;10(7):e0133123. doi: 10.1371/journal.pone.0133123 (PMC4521831; doi:10.1371/journal.pone.0133123)
Supplement: S5 Table — (PDF) [file pone.0133123.s012.pdf]

Table S5.- Linear correlation matrix of root system parameters measured in carnation stem cuttings grown *in vitro*

	RA	RD	RW	ARD	MXR	RWD	RLD	RS
RL	**0.971**	0.788	0.754	-0.224	**0.867**	-0.437	-0.117	-0.300
RA		0.763	0.755	-0.145	**0.836**	-0.420	-0.108	-0.261
RD			0.803	-0.426	0.659	-0.696	*-0.022*	-0.680
RW				-0.362	0.756	-0.328	*-0.038*	-0.631
ARD					-0.342	0.357	*0.021*	0.623
MXR						-0.241	-0.132	-0.265
RWD							-0.109	0.573
RLD								-0.157

Non-significant correlations ($P > 0.05$) are shown in italics. r values larger than 0.81 ($r^2 \sim 0.65$) are indicated in bold.
